# Molecular mechanisms underlying sex and treatment-dependent differences in an animal model of cue-exposure therapy for cocaine relapse prevention

**DOI:** 10.3389/fnins.2024.1425447

**Published:** 2024-08-08

**Authors:** Lucy Peterson, Jonathan Nguyen, Naveed Ghani, Pedro Rodriguez-Echemendia, Hui Qiao, Sun Young Guwn, Heng-Ye Man, Kathleen M. Kantak

**Affiliations:** ^1^Department of Pharmacology, Physiology and Biophysics, Boston University Chobanian and Avedisian School of Medicine, Boston, MA, United States; ^2^Department of Biology, Boston University, Boston, MA, United States; ^3^Department of Psychological and Brain Sciences, Boston University, Boston, MA, United States

**Keywords:** cocaine-cue extinction, cocaine self-administration, environmental enrichment, Org24598, relapse, neuroplasticity, sex differences

## Abstract

Environmental enrichment combined with the glycine transporter-1 inhibitor Org24598 (EE+ORG) during cocaine-cue extinction (EXT) inhibited reacquisition of 1.0 mg/kg cocaine self-administration in male but not female rats in a previous investigation. In this investigation, we determined if this treatment benefit in males required EXT training and ascertained the molecular basis for the observed sex difference in treatment efficacy. Nine groups of male rats trained to self-administer 1.0 mg/kg cocaine or receiving yoked-saline underwent EXT or NoEXT with or without EE and/or ORG. Next, they underwent reacquisition of cocaine self-administration or were sacrificed for molecular analysis of 9 protein targets indicative of neuroplasticity in four brain regions. Two groups of female rats trained to self-administer 1.0 mg/kg cocaine also underwent EXT with or without EE + ORG and were sacrificed for molecular analysis, as above. EE + ORG facilitated the rate of EXT learning in both sexes, and importantly, the therapeutic benefit of EE + ORG for inhibiting cocaine relapse required EXT training. Males were more sensitive than females to neuroplasticity-inducing effects of EE + ORG, which prevented reductions in total GluA1 and PSD95 proteins selectively in basolateral amygdala of male rats trained to self-administer cocaine and receiving EXT. Females were deficient in expression of multiple protein targets, especially after EE + ORG. These included total GluA1 and PSD95 proteins in basolateral amygdala, and total TrkB protein in basolateral amygdala, dorsal hippocampus, and ventromedial prefrontal cortex. Together, these results support the clinical view that sex-specific pharmacological and behavioral treatment approaches may be needed during cue exposure therapy to inhibit cocaine relapse.

## 1 Introduction

Over a decade ago, we developed an animal model of cue exposure therapy to study whether strategies that enhance extinction memory could inhibit relapse to cocaine self-administration to a greater extent than extinction training alone in laboratory animals (Nic Dhonnchadha et al., 2010). In subsequent studies utilizing a second-order schedule of cocaine delivery and cue presentation, male rats treated during cocaine cue extinction training with an optimal dose (3.0 mg/kg) of the glycine transporter-1 (GlyT-1) inhibitor Org24598 (Achat-Mendes et al., 2012) or with brief 4 h periods of environmental enrichment (EE) occurring both 24 h before and immediately after each weekly cocaine-cue extinction training session (Gauthier et al., 2017) reduced reacquisition of 0.3 mg/kg cocaine self-administration over multiple successive daily sessions compared to male rats that received cocaine cue extinction training alone. Critically, when 3.0 mg/kg Org24598 or brief EE exposure was administered to male rats that did not receive cocaine-cue extinction training, these treatments were not protective and rats rapidly relapsed to baseline rates of cocaine self-administration during the reacquisition period. Collectively, these results indicate that cocaine-cue extinction training is a necessary component for inhibiting reacquisition of 0.3 mg/kg cocaine self-administration after Org24598 pretreatment or with brief EE exposure periods. Mechanistically, drugs that inhibit astrocytic GlyT-1 increase synaptic glycine availability at the NR1 subunit of NMDA receptors and enhance memory, including extinction memory (Bergeron et al., 1998; Mao et al., 2009; Shimazaki et al., 2010; Harada et al., 2012). Org24598 is of particular interest because it is a highly specific GlyT-1 inhibitor, with negligible effects on GlyT-2 or receptors such as dopamine, serotonin, and adrenoreceptors (Brown et al., 2001). It also is 600x more potent than sarcosine, another GlyT-1 receptor, as well as forebrain specific (Brown et al., 2001). EE on the other hand interacts with BDNF (Brain Derived Neurotrophic Factor) and its binding at the TrkB receptor as well as with glutamate and its binding at the NR2B subunit of the NMDA receptor to promote synaptic plasticity (Tang et al., 2001; Segovia et al., 2006; Bechara et al., 2014).

As a moderate 0.3 mg/kg unit dose of cocaine was used for self-administration training and testing in the above studies, it was not clear if the individual effects of Org24598 and EE noted above would persist if cocaine intake were of greater magnitude, or if both treatments would need to be combined with cocaine-cue extinction training to be of benefit. Prolonged use of a high, 1.0 mg/kg, cocaine training dose is thought to produce addiction-like behavior (Deroche-Gamonet et al., 2004; Economidou et al., 2009), and thus is likely to be more relevant than a more moderate 0.3 mg/kg cocaine training dose for evaluating the effects of Org24598 and EE for cocaine addiction treatment. This idea was evaluated in male and female rats trained to self-administer 1.0 mg/kg cocaine for a prolonged period (Kantak et al., 2020), and the results were surprising because sex differences were observed. In male rats, 3.0 mg/kg Org24598 + EE combined with cocaine cue extinction training inhibited reacquisition of 1.0 mg/kg cocaine self-administration for multiple successive daily sessions compared to rats that only received cocaine-cue extinction training or received cocaine-cue extinction training combined with just 3.0 mg/kg Org24598 or just EE. In female rats, neither 3.0 nor 7.5 mg/kg Org24598 combined with EE and cocaine-cue extinction training inhibited reacquisition of 1.0 mg/kg cocaine self-administration compared to cocaine-cue extinction training alone. Instead, female rats from each treatment group rapidly relapsed to baseline rates of cocaine self-administration during the reacquisition period. The 7.5 mg/kg dose of Org24598 combined with EE clearly was behaviorally active in female rats in that it facilitated the rate of cocaine-cue extinction learning in a similar manner as the 3.0 mg/kg dose of Org24598 did in male rats, yet it was ineffective in enhancing extinction memory formation or its retrieval for cocaine relapse prevention in the females.

In terms of molecular changes, one past study examined protein targets in male rats trained to self-administer the more moderate 0.3 mg/kg cocaine (Gauthier et al., 2017). We showed that a prolonged history of cocaine self-administration alone reduced the expression of AMPA receptor subunit GluA1 in the basolateral amygdala (BLA) and nucleus accumbens (NAc) and reduced amount of the phosphorylated GluA1 at serine 845 (pSer845GluA1) in the BLA, but not in ventromedial prefrontal cortex (vmPFC) or dorsal hippocampus (DH). Extinction training provided either alone or in combination with EE produced increased expression of pSer845GluA1 in BLA and total GluA1 in all four brain regions of male rats. In a second study examining molecular targets in male rats trained to self-administer 0.3 mg/kg cocaine (Hastings et al., 2020), the expression levels of total TrkB, pTyr816TrkB, total GluA1, pSer845GluA1, and AMPA receptor subunit GluA2 in BLA, NAc, vmPFC, and DH were determined. The pattern of protein expression changes found specifically in rats receiving EE combined with cocaine-cue extinction training in the familiar self-administration context (the only experimental condition that effectively inhibited reacquisition of cocaine self-administration for multiple successive daily sessions), included increased expression of total TrkB in DH, pTyr816TrkB in vmPFC and NAc, and pSer845GluA1 in NAc. Together, these findings suggest an important role for AMPA and TrkB receptors within several brain regions implicated in cocaine-cue extinction (Nic Dhonnchadha and Kantak, 2011) in mediating the beneficial effects of EE for inhibiting a moderate dose (0.3 mg/kg) of self-administered cocaine.

The aim of the present study was to determine if the treatment benefit of Org24598 + EE for cocaine relapse prevention in male rats trained to self-administer a high 1.0 mg/kg dose of cocaine required extinction training and to ascertain if there might be sex differences in the expression levels of several key molecular targets in selected brain regions that might account for the previously observed sex differences in cocaine relapse prevention by this extinction treatment intervention. Two experiments were performed. Experiment 1 was conducted in male rats. It was designed to analyze the effects of EE and Org24598 (ORG) treatments separately and together during cocaine-cue extinction (EXT) or no-extinction (NoEXT) training to extend our earlier behavioral observations with the 1.0 mg/kg cocaine training dose and to understand the molecular basis for the success of this multimodal treatment intervention for cocaine relapse prevention in male rats. Experiment 2 was conducted in female rats also trained to self-administer a high 1.0 mg/kg dose of cocaine. It was designed to determine the molecular changes associated with EXT training alone and EXT training combined with EE + ORG treatment in female rats. Any sex differences in protein expression would be informative for understanding why there were sex differences in the effectiveness of EE + ORG for cocaine relapse prevention. In addition to determining expression levels of TrkB (total and pTyr816) and AMPA receptor subunits GluA1 (total and pSer845) and GluA2, we determined expression levels of other proteins involved in synaptic plasticity, including PSD95, GABA receptor subunit GABA_A_Rα1, and NMDA receptor subunits NR1 and NR2B (Ehlers, 2000; Malinow and Malenka, 2002; Man et al., 2007; Li and Wolf, 2011).

## 2 Materials and methods

### 2.1 Animals

One-hundred and three male (251–275 g) and fourteen female (176–200 g) Wistar rats (WIS/Crl) were 8 weeks old upon arrival from Charles River Laboratories, USA. Rats were housed individually in ventilated-rack cages located in separate climate-controlled colony rooms under a 12 h light/dark cycle (07:30 h on, 21:30 h off). All procedures took place during the light phase. Unless otherwise noted, rats had free access to food and water in their home cages. All procedures complied with the 8th edition of the NIH Guide for Care and Use of Laboratory Animals and were approved by the Boston University Institutional Animal Care and Use Committee.

### 2.2 Drugs

Cocaine hydrochloride (National Institute on Drug Abuse Drug Supply, Bethesda, MD, USA) was dissolved in 0.9% saline containing 3.0 IU heparin/ml of solution. The cocaine training dose for self-administration was 1.0 mg/kg (5.4 mg/ml) infused intravenously (i.v.) at 1.8 ml/min for 0.6 s/100 g body weight. Org24598 (ORG; Tocris Bio-Techne Corporation, Minneapolis, MN, USA) was dissolved in 45% 2-hydroxypropyl-β-cyclodextrin. Doses of ORG (3.0 mg/kg in male rats and 7.5 mg/kg in female rats) or its vehicle (VEH) were injected intraperitoneally (i.p. 1.0 ml/kg) 30-min prior to each extinction or abstinence training session (see Section 2.3.3). Drug dose selections were based on our earlier research findings (Achat-Mendes et al., 2012; Kantak et al., 2020).

### 2.3 Behavioral methods

#### 2.3.1 Lever press training

After acclimation to the animal facility for at least 72 h, rats were food restricted and then trained under a fixed-ratio 1 (FR 1) schedule to rapidly earn 100 food pellets to facilitate later operant lever pressing for cocaine. During food pellet training, depression of either the right or left lever resulted in the delivery of a 45 mg chocolate-flavored pellet (Bio-Serv, Frenchtown, NJ, USA). To maintain motivation for lever pressing during these daily sessions, the 100 food pellets were supplemented with limited amounts of rodent chow in the home cages (8–10 g in male rats and 6–8 g in female rats). After 2–3 days of training, rats typically lever pressed for 100 food pellets within 10–20 min.

#### 2.3.2 Baseline cocaine self-administration sessions

Following lever press training, catheters were implanted into the right jugular vein (Szalay et al., 2013) and post-surgical care and catheter maintenance procedures were implemented (Hastings et al., 2020) as described previously. Catheters were tested periodically for patency either by the ability to aspirate blood or the ability to induce rapid transient loss of muscle tone after i.v. infusion (1.0 mg/0.1 ml) of methohexital sodium (Brevital; JHP Pharmaceuticals, Rochester, MI, USA). The daily (Monday-Friday) 1 h cocaine self-administration or yoked-saline sessions began 1 week after surgery in the self-administration chambers (Szalay et al., 2013), with home cage food restriction during the first few sessions. Each yoked-saline rat received the same number and temporal pattern of infusions and cue light presentations as the cocaine self-administering rat to which it was paired. Assignment of the right or left lever as the active lever was randomly counterbalanced across subjects self-administering cocaine, with the alternate lever designated as the inactive lever. All rats first self-administered cocaine or received yoked-saline under an FR 1 schedule and then under an FR 5 schedule, with a 2-s cue light paired with each cocaine infusion. A maximum of 15 infusions of 1.0 mg/kg cocaine was allowed per 1 h session under FR training schedules to protect against overdose. Once FR 5 responding was reliably maintained, cocaine and yoked-saline delivery and cue presentation transitioned to a second-order [FI 2 min (FR 5: S)] schedule. Under this schedule, every fifth press on active lever (FR 5) during the 2 min fixed-interval (FI) produced a 2-s cue light (S), and the first FR 5 completed after the 2 min FI elapsed produced the 2-s cue light paired with a 1.0 mg/kg cocaine infusion or yoked-saline delivery. Responses on the inactive lever were counted separately but had no scheduled consequences. In addition, white noise (70-db) was presented in the background for the duration of each session. Cocaine self–administration was well–established (35–40 daily sessions) to provide a long cocaine history before extinction or abstinence training began (see Section 2.3.3). Behavioral measures included the number of active lever responses, the number of inactive lever responses, and the daily mg/kg cocaine intake. The last 5 daily training sessions were used to establish baseline performance under the second-order schedule of cocaine delivery.

#### 2.3.3 Extinction and abstinence sessions

Beginning 48 h after the last baseline sessions, lever pressing was either extinguished (extinction sessions) or not extinguished (abstinence sessions). Each extinction (EXT) training session began with the 2 min FI component during which the drug-paired cue light was presented for 2 s after completing each FR 5 on the active lever. However, the anticipated i.v. infusion of cocaine at the end of the initial cue-only component was withheld and rats received an i.v. infusion of saline instead. EXT training with saline infusions in the cocaine-trained rats continued for the remainder of the 1 h session under the FI 2 min (FR 5:S) second-order contingencies. Six EXT training sessions (1 h/week for 6 weeks) were conducted, analogous to clinical protocols utilizing 1 h sessions spaced at weekly intervals (Hofmann et al., 2015; Otto et al., 2016). As a control condition for EXT training, other rats that were trained to self-administer cocaine or receive yoked-saline underwent abstinence (NoEXT) sessions beginning 48 h after the last cocaine or yoked-saline session. During six weekly 1 h NoEXT sessions, levers were retracted in the chambers, no cocaine-associated stimuli (cue light or white noise) were presented, and no infusions were delivered. During EXT and NoEXT sessions, rats also received either EE or NoEE (see Section 2.3.4) and either ORG or VEH (see Section 2.2). Behavioral measures for rats that underwent EXT training included the number of active and inactive lever responses for each of the six weekly sessions under the second-order schedule of saline delivery.

#### 2.3.4 Environmental enrichment sessions

Environmental enrichment (EE) sessions were used in conjunction with EXT and NoEXT sessions and consisted of 4 h periods in the enrichment chambers. As previously described (Gauthier et al., 2017), environmental enrichment took place in a 76 × 46 × 74 cm powder-coated wire cage (Super Pet Inc., Walnut Creek, CA, USA) equipped with two running wheels, three levels of ramps and platforms, movable tunnel structures, and numerous chew toys. Items were changed each week to maintain novelty. Commercial bedding covered the cage floor, and pieces of sweetened cereal were hidden in various locations throughout the cage to encourage foraging. During the self-administration baseline period, rats were acclimated in weekly 15 min periods to the EE chamber and companion animals on two or three occasions to ensure compatibility prior to incorporating the 4 h EE sessions. During EXT or NoEXT sessions in some groups of rats, the 4 h EE sessions were provided both 24 h before and immediately after each of the weekly EXT or NoEXT sessions. Three to four compatible rats were placed together during each of these 4 h EE sessions. The remaining groups of rats remained in their home cages before and after their weekly EXT or NoEXT sessions without receiving EE (NoEE).

#### 2.3.5 Reacquisition of cocaine self-administration

Reacquisition of cocaine self-administration was evaluated beginning 1 week after the last EXT or NoEXT session. During this experimental phase, no treatments were provided. The FI 2 min (FR5: S) second-order contingencies were identical to those established during the baseline cocaine self-administration phase, as described in Section 2.3.2. The reacquisition phase lasted for 15 consecutive daily sessions (Monday–Friday) and behavioral measures included the number of active lever responses, the number of inactive lever responses, and the daily mg/kg cocaine intake.

### 2.4 Molecular methods

#### 2.4.1 Brain tissue lysate preparation

Unanesthetized rats were euthanized by rapid decapitation. Brains were removed and then specific brain regions (BLA, DH, NAc, vmPFC) were cut into 0.5–1 mm sections using a rat brain matrix (RBM-4000C, ASI Instruments, Warren, MI) and razor blades according to published methods (Heffner et al., 1980). Landmarks within each section were compared to the Paxinos rat brain atlas (Paxinos and Watson, 1982) and regions of interest were removed with a cold scalpel. Immediately after dissection, tissues were flash frozen in dry ice-cooled 2-methylbutane. Frozen tissues were homogenized as separate samples. Tissues from the nine groups of males were divided into six cohorts, and tissues from the two groups of females were combined as one cohort, to ensure blinding during experimentation. A homogenate of total brain tissue obtained from a male rat confined to its home cage was analyzed as control tissue for all assays. Protein lysates were prepared for biochemical analysis by first using BCA analysis to normalize all protein sample concentrations. Then proteins were denatured by boiling in 2x Laemmli sample reducing buffer (4% SDS, 10% 2-mercaptoethanol, 20% glycerol, 0.004% bromophenol blue, 0.125M Tris HCl) for 10 min at 95°C.

#### 2.4.2 Immunoblotting

To immunoblot, 40 μg of each protein homogenate was subjected to SDS-PAGE. Quadruplets of each sample were run to allow for immunoblotting with multiple sets of antibodies. After electrophoresis, proteins were transferred to a PVDF membrane and blocked in 10% milk in 1x TBST (20 mM Tris, 150 mM NaCl, 0.1% w/v Tween-20). One membrane was probed with antibodies against GluA1 (Cell Signaling, #13185), PhosphoSer845-GluA1 (MilliporeSigma, #AB5849), and Tubulin (MilliporeSigma, #AB9354) as a protein loading control. Another membrane was probed with antibodies against TrkB (BD Biosciences, #610101), PhosphoTyr816-TrkB (MilliporeSigma, #ABN1381), Tubulin. The third membrane was probed with antibodies against GluA2 (MilliporeSigma, #MAB397), NR1 (Abcam, #ab17345), and Tubulin. The fourth membrane was probed with antibodies against GABA_A_Rα1 (Biolegend, #11362), NR2B (Sigma Aldrich, #454585), PSD95 (Cell Signaling, #2507), and Tubulin. Membranes were then incubated in the appropriate species secondary antibodies conjugated to horseradish peroxidase (HRP) (#62-6520, Invitrogen) followed by the ECL substrate to image the protein of interest.

### 2.5 Experimental design

#### 2.5.1 Experiment 1: behavioral and molecular effects in male rats

**Figure 1:**
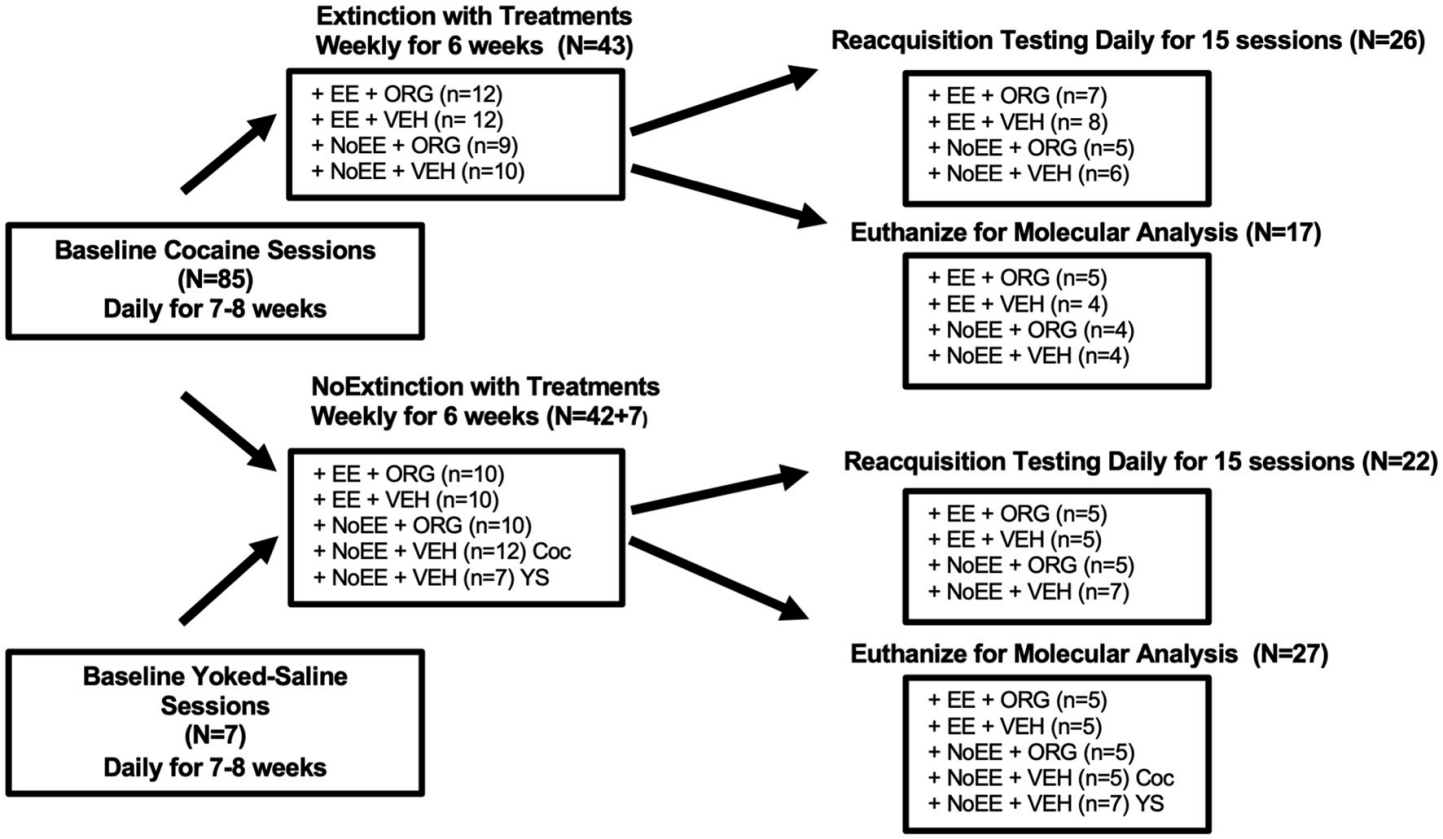
Experimental design for the behavioral and molecular analyses in male rats. Boxes show the number of subjects per group during the baseline, extinction and reacquisition/molecular phases of testing. Rats were trained to self-administer 1.0 mg/kg cocaine (Coc) or receive yoked-saline (YS). Treatments that were administered only during the extinction phase consisted of extinction (EXT) or no extinction (NoEXT) training, environmental enrichment (EE) or no environmental enrichment (NoEE) exposure, and Org24598 (ORG) or vehicle (VEH) administration. After the extinction phase, rats either underwent the reacquisition phase or were euthanized for molecular analyses.

Of the 103 male rats, five did not acquire cocaine self-administration during baseline sessions and six lost catheter patency before the end of the experiment. As illustrated in Figure 1, the 92 male rats that completed the experiment were either trained to self-administer 1.0 mg/kg cocaine (*N* = 85) or to passively receive yoked-saline (*N* = 7). Upon completion of baseline self-administration training, 43 of the 85 cocaine rats were assigned randomly to receive the weekly EXT training sessions, combined with either EE + ORG (*n* = 12), EE + VEH (*n* = 12), NoEE + ORG (*n* = 9), or NoEE + VEH (*n* = 10). The remaining 42 of the 85 cocaine rats that completed baseline self-administration training were assigned randomly to receive weekly NoEXT sessions, combined with either EE + ORG (*n* = 10), EE + VEH (*n* = 10), NoEE + ORG (*n* = 10), or NoEE + VEH (*n* = 12). The seven yoked-saline rats were paired with cocaine rats that received NoEXT + NoEE + VEH. Upon completion of the extinction treatment protocol, rats either underwent reacquisition testing (*n* = 5–8 per treatment group) or were euthanized by live decapitation for molecular measures (*n* = 4–5 per treatment group). Upon completion of the NoEXT treatment protocol, rats also either underwent reacquisition testing (*n* = 5–7 per treatment group) or were euthanized by live decapitation.

#### 2.5.2 Experiment 2: behavioral and molecular effects in female rats

Following baseline training in the 14 female rats, two did not acquire cocaine self-administration. The remaining 12 female rats were then assigned randomly to receive six weekly EXT training sessions combined with EE + ORG (*n* = 6) or with NoEE + VEH (*n* = 6). Upon completion of the extinction treatment protocol, rats were euthanized by live decapitation for molecular measures. To examine additional groups of female rats as was done for the males for the behavioral and molecular analyses would have been superfluous because our past research (Kantak et al., 2020) showed that there was no benefit of EE + ORG treatment for cocaine relapse prevention in the females as there was in the males. Thus, there was no need to determine if EXT training was required for a treatment benefit in females by examining a female NoEXT control group and no need to examine the effects of EE alone or ORG alone with EXT training in females. Therefore, for determining sex differences in protein expression, we focused on the most important groups: EXT + NoEE + VEH (extinction training alone) and EXT + EE + ORG (extinction training with the most efficacious treatment in males) to compare protein expression in males and females.

### 2.6 Statistical analysis

All data were analyzed using GraphPad Prism 10.0 statistical software. Normality was checked for each analysis of variance (ANOVA) using quantile–quantile (Q-Q) plots. Normally distributed data have Q-Q plots with residuals that fall along an approximately straight line at a 45-degree angle. For all ANOVAs, the majority of points fell along the reference line. There were only a few points that deviated from the reference line at the extreme ends of the distribution, suggesting that data were reasonably normally distributed for each dataset (Thode, 2002). For one-factor ANOVAs, equal variance was checked using the Brown-Forsythe test, with individual subject data transformed to the square root of × prior to the analysis if this test was significant. In the two-factor and three-factor repeated measures ANOVAs, the Greenhouse-Geisser test for sphericity was used to check for equal variance, with the degrees of freedom (df) and probability (*p*) values adjusted if this test was significant. Adjustments were necessary for most session number main effects in the two-factor and three-factor repeated measures ANOVAs (denoted in the text by indicating the df and *p*-values that were adjusted) and for some group differences in protein expression in the one-factor ANOVAs (denoted in the text by indicating that the data were transformed).

#### 2.6.1 Behavioral measurements

The four groups of male rats in experiment 1 that had received NoEXT sessions were combined into a single NoEXT control group, as there were no significant group differences in the behavior of these rats during cocaine self-administration baseline and reacquisition sessions (Supplementary Tables 1, 2). Due to 3- to 10-fold (males) and 5- to 9-fold (females) differences in baseline active lever responses among individual rats within each of the treatment and control groups in experiments 1 and 2, active lever responses during the weekly extinction sessions and the daily reacquisition sessions were expressed as percent of baseline responses prior to analysis. Each behavior was analyzed by a 1-factor (group) analysis of variance (ANOVA) or a 2-factor (group × session number) repeated measures ANOVA (repeated on the session number factor). The four groups of male rats that had received EXT sessions (EXT + NoEE + VEH, EXT + EE + VEH, EXT + NoEE + ORG, and EXT + EE + ORG) and the two groups of female rats that had received EXT sessions (EXT + NoEE + VEH and EXT + EE + ORG) were compared to the NoEXT control group in separate ANOVAs. Following significant ANOVA factors, Dunnett or Tukey *post-hoc* tests were used to compare group and session number differences.

#### 2.6.2 Molecular measurements

To control for variability in total protein between samples, all samples for western blot analysis were loaded at equal concentration, as determined via Bicinchoninic (BCA) assay (ThermoScientific). Some samples (72 of 1,584) with abnormally low protein concentration did not have enough volume to be used for all protein probes (see starting group sizes specified in Figure 1 as compared to final group sizes specified in Tables 1–4). To control for between-assay variability in the Western blots, the band intensity for each of the nine molecular targets was normalized first to each rat's own tubulin value (protein loading control). These values within each cohort were next normalized to contemporaneously assayed home cage control tissue (tissue loading control). Lastly, these values from rats that underwent extinction training were normalized to the no-extinction control group (behavioral loading control). To analyze molecular changes associated with extinction training in male rats with only a cocaine history, relative protein expression in the EXT + NoEE + VEH group was compared to the Yoked-Saline and NoEXT control groups by 1-factor (group) ANOVA. To analyze molecular changes associated with the different treatments in male rats, one-factor (group) ANOVA was used to compare relative protein expression from each brain region in the EXT + NoEE + VEH, EXT + EE + VEH, EXT + NoEE + ORG, and EXT + EE + ORG treatment groups to the NoEXT control group. To analyze treatment group differences in female rats, one-factor (group) ANOVA was used to compare relative protein expression in the EXT + NoEE + VEH and EXT + EE + ORG treatment groups to the male NoEXT control group. To analyze sex differences in the molecular changes produced by EXT + EE + ORG, each molecular target was analyzed by two-factor ANOVA (group × sex). The male and female EXT + NoEE + VEH and EXT + EE + ORG treatment groups were compared in these analyses. Following significant ANOVA factors, Dunnett (after 1-factor ANOVAs) and Tukey (after 2-factor ANOVAs) *post-hoc* tests were used to compare group and sex differences.

**Table 1:** Molecular changes in basolateral amygdala in male and female rats.

**Sex**	**Treatment**	**Total TrkB**	**pTyr816TrkB**	**GABAARα1**	**PSD95**	
Male	YS control	0.99 ± 0.18 (6)	1.03 ± 0.19 (6)	1.11 ± 0.14 (6)	1.40 ± 0.49 (6)	
	NoEXT control	1.00 ± 0.08 (20)	1.00 ± 0.09 (20)	1.00 ± 0.10 (20)	1.00 ± 0.07 (20)	
	EXT + NoEE + VEH	0.89 ± 0.25 (4)	0.87 ± 0.14 (4)	0.63 ± 012 (4)	**0.57** **±0.04** (4)^**b**^	
	EXT + EE + ORG	1.35 ± 0.16 (5)	1.77 ± 0.58 (5)	0.92 ± 0.12 (5)	1.27 ± 0.11 (5)	
	EXT + EE + VEH	1.19 ± 0.24 (4)	1.61 ± 0.39 (4)	0.67 ± 0.16 (4)	0.87 ± 0.23 (4)	
	EXT + NoEE + ORG	1.06 ± 0.05 (4)	1.07 ± 0.21 (4)	1.00 ±0.17 (4)	1.34 ± 0.03 (4)	
Female	EXT + NoEE + VEH	**0.50** **±0.12** (6)^**b**^	1.38 ± 0.72 (6)	1.06 ± 0.14 (6)	**0.25** **±0.03** (6)^**b**^	
	EXT + EE + ORG	**0.60** **±0.16** (6)^**b**^	1.05 ± 0.17 (6)	0.70 ± 0.16 (6)	**0.22** **±0.05** (6)^**b**^	
**Sex**	**Treatment**	**Total GluA1**	**pSer845GluA1**	**GluA2**	**NR1**	**NR2B**
Male	YS control	1.24 ± 0.27 (6)	0.62 ± 0.24 (6)	0.81 ± 0.11 (6)	3.03 ± 1.31 (6)	1.45 ± 0.29 (6)
	NoEXT control	1.00 ± 0.06 (20)	1.00 ± 0.06 (20)	1.00 ± 0.07 (20)	1.00 ± 0.09 (20)	1.00 ± 0.11 (20)
	EXT + NoEE + VEH	**0.50** **±0.16** (4)^**a, b**^	0.83 ± 0.08 (4)	0.60 ± 0.18 (4)	**0.82** **±0.23** (4)^**a**^	**0.56** **±0.21** (4)^**a**^
	EXT + EE + ORG	0.74 ± 0.04 (5)	1.07 ± 0.31 (5)	0.95 ± 0.14 (5)	1.13 ± 0.18 (5)	1.26 ± 0.23 (5)
	EXT + EE + VEH	0.72 ± 0.18 (4)	1.05 ± 0.08 (4)	0.71 ± 0.12 (4)	0.73 ± 0.19 (4)	0.97 ± 0.33 (4)
	EXT + NoEE + ORG	1.27 ± 0.37 (4)	1.05 ± 0.08 (4)	0.98 ± 0.13 (4)	1.02 ± 0.19 (4)	1.14 ± 0.19 (4)
Female	EXT + NoEE + VEH	**0.55** **±0.07** (6)^**b**^	1.03 ± 0.13 (6)	0.85 ± 0.09 (6)	0.76 ± 0.09 (6)	0.64 ± 0.06 (6)
	EXT + EE + ORG	**0.38** **±0.05** (6)^**b**^	0.74 ± 0.11 (6)	**0.59** **±0.11** (6)^**b**^	**0.40** **±0.10** (6)^**b**^	**0.48** **±0.10** (6)^**b**^

Values are the mean ± s.e.m. The number in the parentheses indicate treatment group size for these analyses. Bolded values are statistically significant as compared to either YS or NoEXT group.

^a^ps ≤ 0.05 compared to the Yoked-Saline (YS) control.

^b^ps ≤ 0.05 compared to the No-Extinction (NoEXT) control.

**Table 2:** Molecular changes in dorsal hippocampus in male and female rats.

**Sex**	**Treatment**	**Total GluA1**	**pSer845GluA1**	**GluA2**	**NR1**	**NR2B**
Male	Yoked-Saline	1.49 ± 0.40 (7)	0.98 ± 0.30 (7)	1.40 ± 0.41 (7)	1.15 ± 0.25 (7)	1.62 ± 0.37 (7)
	NoEXT	1.00 ± 0.11 (20)	1.00 ± 0.06 (19)	1.00 ± 0.06 (19)	1.00 ± 0.09 (20)	1.00 ± 0.14 (20)
	EXT + NoEE + VEH	1.40 ± 0.18 (4)	1.26 ± 0.21 (4)	0.93 ± 0.19 (4)	1.14 ± 0.19 (4)	1.03 ± 0.34 (4)
	EXT + EE + ORG	1.00 ± 0.36 (5)	0.80 ± 0.18 (5)	0.78 ± 0.25 (5)	1.04 ± 0.40 (5)	1.04 ± 0.59 (5)
	EXT + EE + VEH	1.21 ± 0.28 (4)	0.99 ± 0.20 (4)	0.85 ± 0.35 (4)	1.21 ± 0.35 (4)	0.90 ± 0.24 (4)
	EXT + NoEE + ORG	0.64 ± 0.29 (4)	0.86 ± 0.30 (4)	0.90 ± 0.23 (4)	0.89 ± 0.13 (4)	0.64 ± 0.14 (4)
Female	EXT + NoEE + VEH	**4.05** **±1.18** (6)^**a**^	**0.25** **±0.07** (6)^**a**^	1.13 ± 0.20 (6)	0.71 ± 0.15 (6)	1.17 ± 0.38 (6)
	EXT + EE + ORG	1.63 ± 0.58 (6)	**0.45** **±1.18** (6)^**a**^	1.37 ± 0.25 (6)	**0.49** **±0.21** (6)^**a**^	0.41 ± 0.24 (6)
**Sex**	**Treatment**	**Total TrkB**	**pTyr816TrkB**	**GABAAR**α**1**	**PSD95**	
Male	Yoked-Saline	1.24 ± 0.13 (7)	1.49 ± 0.82 (7)	0.87 ± 0.22 (7)	1.90 ± 0.57 (7)	
	NoEXT	1.00 ± 0.06 (20)	1.00 ± 0.16 (20)	1.00 ± 0.06 (19)	1.00 ± 0.09 (20)	
	EXT + NoEE + VEH	1.01 ± 0.24 (4)	0.79 ± 0.15 (4)	0.79 ± 0.14 (4)	1.04 ± 0.46 (4)	
	EXT + EE + ORG	1.33 ± 0.25 (5)	0.86 ± 0.18 (5)	1.48 ± 0.63 (5)	1.36 ± 0.70 (5)	
	EXT + EE + VEH	0.86 ± 0.07 (4)	0.78 ± 0.09 (4)	1.08 ± 0.34 (4)	1.11 ± 0.28 (4)	
	EXT + NoEE + ORG	1.12 ± 0.24 (4)	0.56 ± 0.23 (4)	1.23 ± 0.17 (4)	1.36 ± 0.59 (4)	
Female	EXT + NoEE + VEH	**1.65** **±0.31** (6)^**a**^	1.04 ± 0.23 (6)	**1.76** **±0.18** (6)^**a**^	0.75 ± 0.30 (6)	
	EXT + EE + ORG	0.77 ± 0.13 (6)	1.03 ± 0.38 (6)	**3.47** **±0.61** (6)^**a**^	**0.25** **±0.14** (6)^**a**^	

Values are the mean ± s.e.m. The number in the parentheses indicate treatment group size for these analyses. No significant differences were detected compared to the Yoked-Saline (YS) control. Bolded values are statistically significant as compared to the NoEXT group.

^a^ps ≤ 0.05 compared to the No-Extinction (NoEXT) control.

**Table 3:** Molecular changes in ventromedial prefrontal cortex in male and female rats.

**Sex**	**Treatment**	**Total GluA1**	**pSer845GluA1**	**GluA2**	**NR1**	**NR2B**
Male	YS control	1.01 ± 0.41 (7)	1.18 ± 0.24 (6)	1.69 ± 0.45 (6)	1.58 ± 0.22 (7)	1.92 ± 0.53 (7)
	NoEXT control	1.00 ± 0.10 (20)	1.00 ± 0.11 (20)	1.00 ± 0.09 (20)	1.00 ± 0.12 (20)	1.00 ± 0.08 (20)
	EXT + NoEE + VEH	0.75 ± 0.19 (4)	0.81 ± 0.22 (4)	0.95 ± 0.31 (4)	0.94 ± 0.48 (4)	1.05 ± 0.28 (4)
	EXT + EE + ORG	0.93 ± 0.21 (5)	1.44 ± 0.29 (5)	1.13 ± 0.27 (5)	0.96 ± 0.23 (5)	0.89 ± 0.18 (5)
	EXT + EE + VEH	0.96 ± 0.24 (4)	1.58 ± 0.47 (4)	1.24 ± 0.30 (4)	1.04 ± 0.20 (4)	1.54 ± 0.43 (4)
	EXT + NoEE + ORG	0.52 ± 0.11 (4)	0.89 ± 0.26 (4)	0.59 ± 0.10 (4)	0.73 ± 0.19 (4)	1.07 ± 0.47 (4)
Female	EXT + NoEE + VEH	0.75 ± 0.26 (6)	0.51 ± 0.20 (6)	**0.23** **±0.06 (6)**^**a**^	**0.13** **±0.04 (6)**^**a**^	**0.62** **±0.13 (6)**^**a**^
	EXT + EE + ORG	0.69 ± 0.18 (6)	0.76 ± 0.22 (6)	**0.25** **±0.04 (6)**^**a**^	**0.20** **±0.03(6)** ^**a**^	**0.59** **±0.13 (6)**^**a**^
**Sex**	**Treatment**	**Total TrkB**	**pTyr816TrkB**	**GABAAR**α**1**	**PSD95**	
Male	YS control	1.92 ± 0.53 (7)	0.84 ± 0.19 (6)	1.36 ± 0.18 (6)	1.40 ± 0.29 (5)	
	NoEXT control	1.00 ± 0.08 (20)	1.00 ± 0.11 (20)	1.00 ± 0.06 (20)	1.00 ± 0.12 (20)	
	EXT + NoEE + VEH	1.05 ± 0.28 (4)	1.19 ± 0.23 (4)	1.00 ± 0.21 (4)	0.78 ± 0.19 (4)	
	EXT + EE + ORG	0.89 ± 0.18 (5)	0.97 ± 0.21 (5)	0.97 ± 0.07 (5)	1.73 ± 0.44 (5)	
	EXT + EE + VEH	1.54 ± 0.43 (4)	0.84 ± 0.16 (4)	1.03 ± 0.07 (4)	1.04 ± 0.27 (4)	
	EXT + NoEE + ORG	1.07 ± 0.47 (4)	0.68 ± 0.18 (4)	0.73 ± 0.16 (4)	0.74 ± 0.09 (4)	
Female	EXT + NoEE + VEH	**0.62** **±0.13 (6)**^**a**^	0.77 ± 0.02 (6)	0.73 ± 0.18 (6)	**0.21** **±0.05 (6)**^**a**^	
	EXT + EE + ORG	**0.59** **±0.13 (6)**^**a**^	0.73 ± 0.10 (6)	0.73 ± 0.12 (6)	**0.17** **±0.03 (6)**^**a**^	

Values are the mean ± s.e.m. The number in the parentheses indicate treatment group size for these analyses. No significant differences were detected compared to the Yoked-Saline (YS) control. Bolded values are statistically significant as compared to the NoEXT group.

^a^ps ≤ 0.05 compared to the No-Extinction (NoEXT) control.

**Table 4:** Molecular changes in nucleus accumbens in male and female rats.

**Sex**	**Treatment**	**Total GluA1**	**pSer845GluA1**	**GluA2**	**NR1**	**NR2B**
Male	Yoked-Saline	1.43 ± 0.28 (5)	2.10 ± 0.57 (5)	3.24 ± 1.54 (5)	2.95 ± 1.10 (5)	0.74 ± 0.14 (5)
	NoEXT	1.00 ± 0.13 (15)	1.00 ± 0.17 (16)	1.00 ± 0.05 (15)	1.00 ± 0.05 (16)	1.00 ± 0.08 (16)
	EXT + NoEE + VEH	0.57 ± 0.21 (3)	**0.52** **±0.32 (3)**^**a**^	0.83 ± 0.10 (3)	**0.93 ± 0.18 (3)** ^**a**^	0.70 ± 0.16 (3)
	EXT + EE + ORG	0.80 ± 0.29 (4)	0.89 ± 0.30 (4)	0.89 ± 0.22 (4)	0.86 ± 0.20 (4)	0.89 ± 0.24 (4)
	EXT + EE + VEH	0.81 ± 0.18 (3)	1.27 ± 0.18 (3)	0.75 ± 0.20 (3)	0.83 ± 0.31 (3)	0.61 ± 0.03 (3)
	EXT + NoEE + ORG	1.52 ± 0.44 (3)	1.28 ± 0.10 (3)	0.92 ± 0.12 (3)	1.27 ± 0.18 (3)	0.97 ± 0.15 (3)
Female	EXT + NoEE + VEH	2.90± 1.37 (6)	0.72 ± 0.33 (6)	**0.64** **±0.11 (6)**^**b**^	**11.01** **±1.67(6)**^**b**^	1.67 ± 0.33 (6)
	EXT + EE + ORG	**4.24** **±1.92 (6)**^**b**^	1.39 ± 0.51 (6)	**0.47** **±0.07 (6)**^**b**^	**11.20** **±2.70 (6)**^**b**^	2.67 1.25 (6)
**Sex**	**Treatment**	**Total TrkB**	**pTyr816TrkB**	**GABAAR**α**1**	**PSD95**	
Male	Yoked-Saline	2.08 ± 0.06 (5)	0.55 ± 0.06 (5)	6.78 ± 2.79 (5)	3.81 ± 2.42 (5)	
	NoEXT	1.00 ± 0.07 (16)	1.00 ± 0.08 (16)	1.00 ± 0.09 (15)	1.00 ± 0.11 (16)	
	EXT + NoEE + VEH	**0.51** **±0.21 (3)**^**a**^	0.61 ± 0.13 (3)	**0.58** **±0.37 (3)**^**a**^	1.13 ± 0.42 (3)	
	EXT + EE + ORG	1.41 ± 0.42 (4)	1.20 ± 0.68 (4)	1.48 ± 0.87 (4)	1.03 ± 0.21 (4)	
	EXT + EE + VEH	1.13 ± 0.35 (3)	0.46 ± 0.17 (3)	0.64 ± 0.20 (3)	0.84 ± 0.14 (3)	
	EXT + NoEE + ORG	0.99 ± 0.18 (3)	0.75 ± 0.17 (3)	0.83 ± 0.07 (3)	1.41 ± 0.24 (3)	
Female	EXT + NoEE + VEH	1.16 ± 0.26 (6)	1.70 ± 0.29 (6)	**11.70** **±1.55** (6)^**b**^	0.83 ± 0.10 (6)	
	EXT + EE + ORG	0.67 ± 0.34 (6)	1.04 ± 0.25 (6)	**6.71** **±1.86** (6)^**b**^	**0.53** **±0.08** (6)^**b**^	

Values are the mean ± s.e.m. The number in the parentheses indicate treatment group size for these analyses. Bolded values are statistically significant as compared to either YS or NoEXT group.

^a^ps ≤ 0.05 compared to the Yoked-Saline (YS) control.

^b^ps ≤ 0.05 compared to the No-Extinction (NoEXT) control.

## 3 Results

### 3.1 Experiment 1: behavioral and molecular effects in male rats

#### 3.1.1 Baseline cocaine self-administration sessions

**Figure 2:**
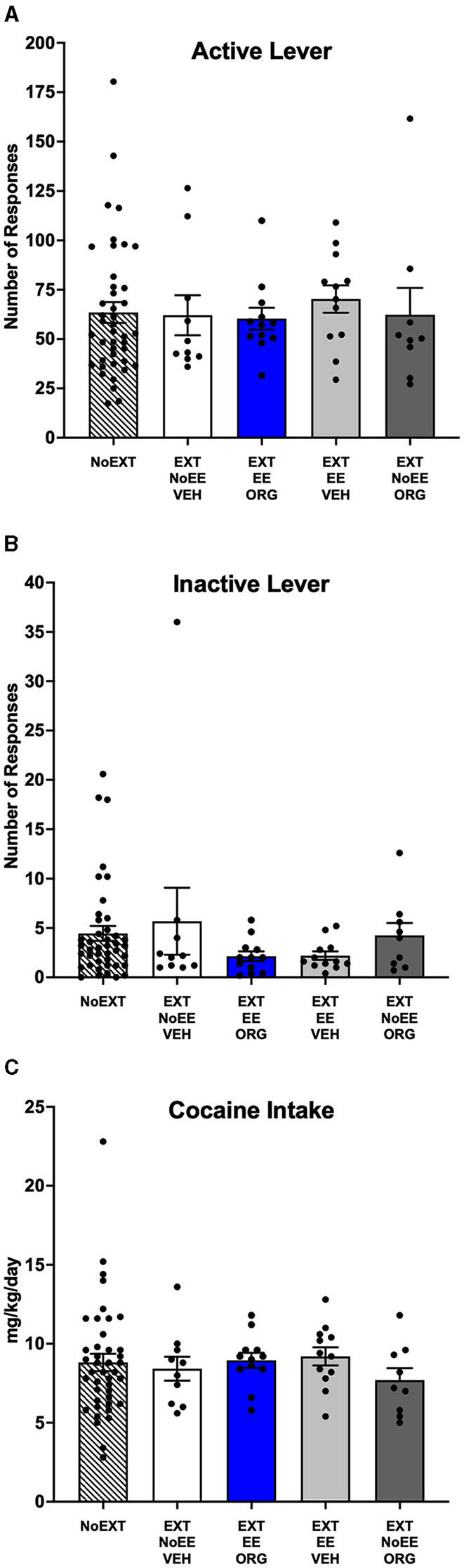
Baseline behavior in male rats. Groups consisted of rats who during the subsequent extinction phase received no extinction training (NoEXT control, *n* = 42) or received cocaine-cue extinction training with no-added treatment (EXT + NoEE + VEH; *n* =12), with EE treatment combined with ORG (EXT + EE + ORG; *n* =12), with EE treatment alone (EXT + EE + VEH; *n* =12), and with ORG treatment alone (EXT + NoEE + ORG; *n* =9). Values are Individual data points and the mean ± s.e.m. for the number of active lever responses **(A)**, the number of inactive lever responses **(B)**, and the daily mg/kg cocaine intake **(C)** averaged over the last five sessions of baseline self-administration training. There were no significant between group differences for any of the baseline measures in male rats.

Cocaine self-administration behavior at baseline was similar among the five groups of male rats prior to initiating treatments. For the number of active lever responses (Figure 2A), there were no significant between-group differences [*F*_(4, 80)_ = 0.17, *p* = 0.95]. There was a similar outcome for the number of inactive lever responses (Figure 2B) in that there were no significant between-group differences [*F*_(4, 80)_ = 1.07, *p* = 0.38]. The overall daily cocaine intake averaged 8.5 mg/kg/day during baseline (Figure 2C), with no significant between-group differences as well [*F*_(4, 80)_ = 0.41, *p* = 0.80].

#### 3.1.2 Extinction sessions

**Figure 3:**
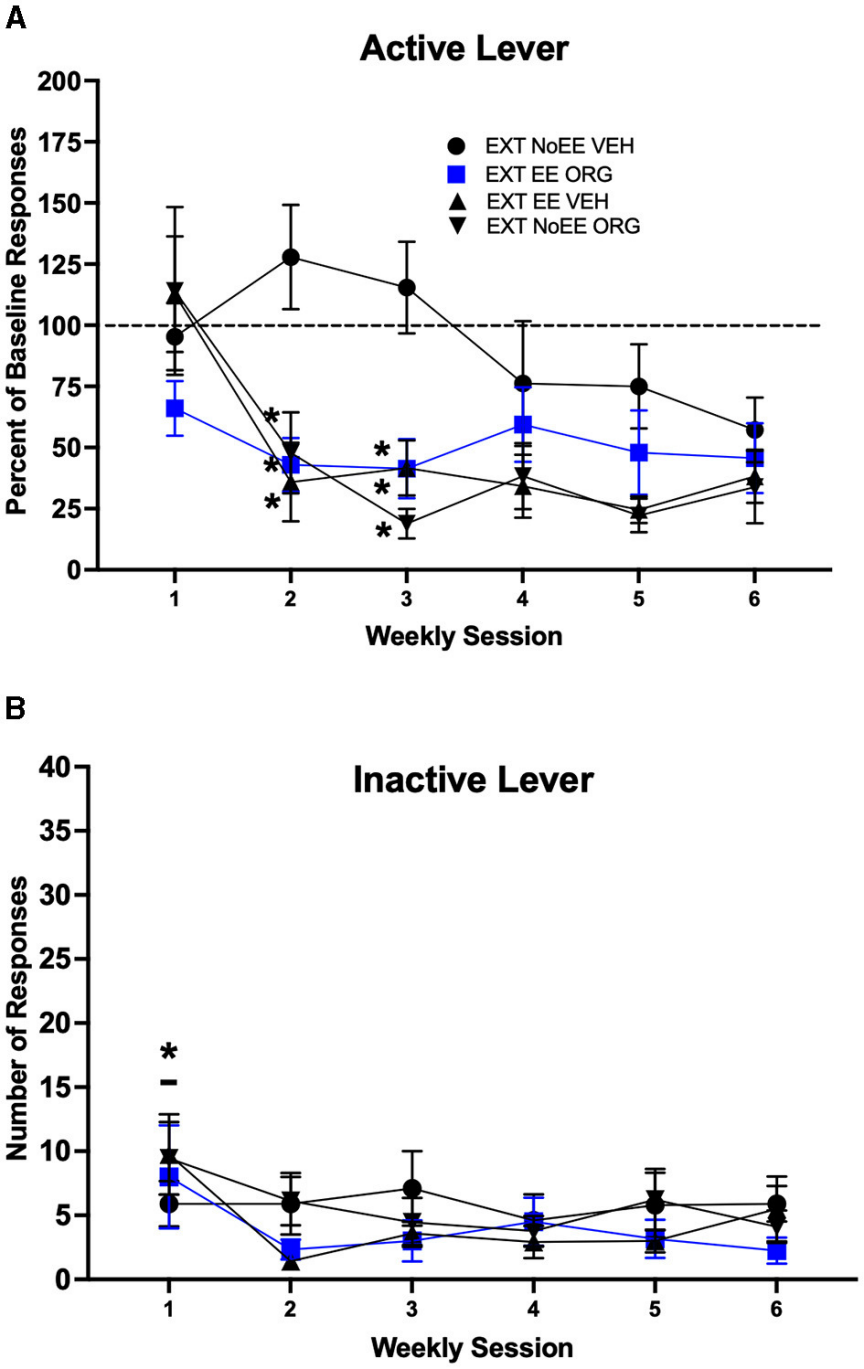
Extinction behavior in male rats. Groups consisted of rats who during the extinction phase received cocaine-cue extinction training with no-added treatment (EXT + NoEE + VEH; *n* =12), with EE treatment combined with ORG (EXT + EE + ORG; *n* =12), with EE treatment alone (EXT + EE + VEH; *n* =12), and with ORG treatment alone (EXT + NoEE + ORG; *n* =9). Values are the mean ± s.e.m. for the percent of baseline active lever responses **(A)** and the number of inactive lever responses **(B)** for each of the 6 weekly extinction training sessions. *ps ≤ 0.003 compared to the EXT + NoEE + VEH group. *p = 0.003 compared to weeks 2–6 overall.

The EXT + EE + ORG, EXT + EE + VEH, and EXT + NoEE + ORG treatments facilitated cocaine-cue extinction learning compared to extinction training alone (EXT + NoEE + VEH). Active lever responding (Figure 3A) declined across the course of extinction training [main effect of session number; *F*_(3.5, 136.1)_ = 8.7, *p* = 0.0001; adjusted df and *p*-values], with significantly less responding during weeks 2–6 compared to week 1 overall (ps ≤ 0.003). However, the magnitude of decline in active lever responding across the course of extinction training was different among the four treatment groups [group × session number interaction; *F*_(15, 195)_ = 2.5, *p* = 0.002]. Rats that received EXT + EE + ORG, EXT + EE + VEH or EXT + NoEE + ORG made fewer active lever responses than rats that received EXT + NoEE + VEH during weeks 2–3 of extinction training (ps ≤ 0.003). Groups did not differ during weeks 4–6 of extinction training. Inactive lever responses (Figure 3B) remained at low levels in all groups across the course of extinction training, but there was a significant main effect of session number [*F*_(2.9, 112.6)_ = 3.4, *p* = 0.023; adjusted df and *p*-values]. Rats in general made more inactive lever responses during week 1 of extinction training compared to weeks 2–6 (ps ≤ 0.02).

#### 3.1.3 Reacquisition of cocaine self-administration sessions

**Figure 4:**
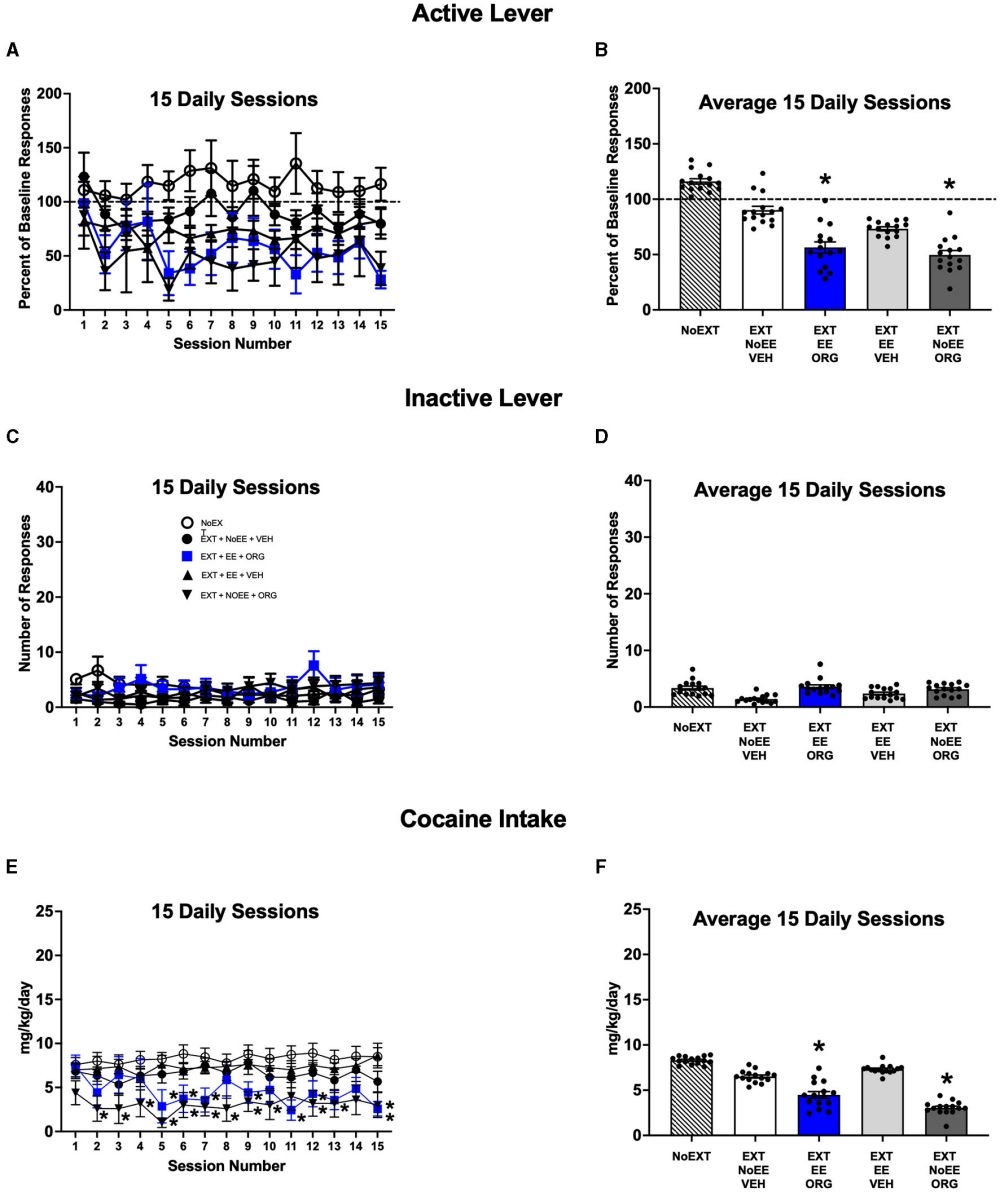
Reacquisition behavior in male rats. Groups consisted of rats who during the preceding extinction phase received no extinction training (NoEXT control, *n* =42) or received cocaine-cue extinction training with no-added treatment (EXT + NoEE + VEH; *n* =12), with EE treatment combined with ORG (EXT + EE + ORG; *n* =12), with EE treatment alone (EXT + EE + VEH; *n* =12), and with ORG treatment alone (EXT + NoEE + ORG; *n* =9). Values are Individual data points and/or the mean ± s.e.m. for the percent of baseline active lever responses for each of the 15 daily sessions **(A)** or averaged over the 15 daily sessions **(B)**, the number of inactive lever responses for each of the 15 daily sessions **(C)** or averaged over the 15 daily sessions **(D)**, and the daily mg/kg cocaine intake for each of the 15 daily sessions **(E)** or averaged over the 15 daily sessions **(F)**. *ps ≤ 0.05 compared to the NoEXT control group.

The EE + ORG and NoEE + ORG treatments that were provided during EXT inhibited subsequent reacquisition of cocaine self-administration in male rats. The magnitude of active lever responding during daily reacquisition sessions (Figures 4A, B) was different among the treatment groups [main effect of group; *F*_(4, 43)_ = 3.2, *p* = 0.02] Active lever responding was significantly lower overall in the EXT + EE + ORG (*p* = 0.039) and EXT + NoEE + ORG (*p* = 0.046) treatment groups compared to the NoEXT control group. The differences in these treatment groups did not depend on session number during the reacquisition period [group X session number interaction; *F*_(56, 602)_ = 0.8, *p* = 0.92]. The EXT+EE+VEH (*p* = 0.17) and EXT + NoEE + VEH (*p* = 0.70) treatment groups did not differ from the NoEXT control group. These latter groups and the NoEXT control group returned to baseline levels of cocaine self-administration responding within the first few reacquisition sessions. The number of inactive lever responses did not differ by group (*p* = 0.61) or session number (*p* = 0.78) or their interaction (*p* = 0.24) during reacquisition sessions (Figures 4C, D). The daily mg/kg cocaine intake during reacquisition sessions (Figures 4E, F) also was different among the treatment groups [main effect of group; *F*_(4, 43)_ = 3.1, *p* = 0.025]. Cocaine intake was significantly lower in the EXT + EE + ORG (*p* = 0.045) and EXT + NoEE + ORG (*p* = 0.019) treatment groups compared to the NoEXT control group. The EXT + EE + VEH (*p* = 0.93) and EXT + NoEE + VEH (*p* = 0.71) treatment groups did not differ from the NoEXT control group. However, the reduction in cocaine intake was different across the 15 daily reacquisition sessions [group X session number interaction; *F*_(56, 602)_ = 1.6, *p* = 0.006]. Cocaine intake in the EXT + EE + ORG treatment group was significantly lower than the NoEXT control group on sessions 5–7, 9, 11–13, and 15 (ps ≤ 0.001–0.05). In the EXT + NoEE + ORG treatment group, cocaine intake was significantly lower than the NoEXT control group on sessions 2–10, 12, and 15 (ps ≤ 0.001–0.05). There were no differences in cocaine intake in the EXT + EE + VEH and EXT + NoEE + VEH treatment groups compared to the NoEXT control group on any of the 15 cocaine reacquisition sessions.

#### 3.1.4 Molecular changes

##### 3.1.4.1 Basolateral amygdala

In analyses of male rats comparing the EXT + NoEE + VEH group (rats with cocaine and extinction histories only) to the two control groups (the Yoked Saline control group with no cocaine, extinction, and treatment histories and the NoEXT control group with cocaine and treatment histories but no extinction history) there were group differences in the relative expression of total GluA1 [*F*_(2, 27)_ = 5.1, *p* = 0.01; data transformed], NR1 [*F*_(2, 27)_ = 4.6, *p* = 0.02; data transformed], and NR2B [*F*_(2, 27)_ = 3.5, *p* = 0.046; data transformed]. As shown in Table 1, Dunnett *post-hoc* testing revealed that total GluA1 expression was significantly lower in the EXT + NoEE + VEH group of male rats compared to the Yoked Saline (*p* = 0.01) and NoEXT (*p* = 0.02) control groups. NR1 (*p* = 0.03) and NR2B (*p* = 0.03) expression were significantly lower in the EXT + NoEE + VEH group of male rats compared to the Yoked Saline control group only. In the analyses to assess the effects of treatment in the four groups of male rats receiving EXT training (EXT + NoEE + VEH, EXT + EE + ORG, EXT + EE + VEH, and EXT + NoEE + ORG) relative to the NoEXT control group, there were group differences in the relative expression of total GluA1 [*F*_(4, 32)_ = 4.1, *p* = 0.01; data transformed] and PSD95 [*F*_(4, 32)_ = 5.2, *p* = 0.003; data transformed]. Table 1 shows that following Dunnett *post-hoc* testing, total GluA1 expression was significantly lower in EXT + NoEE + VEH group of male rats compared to the NoEXT control group (*p* = 0.01) and that groups receiving EXT + EE + ORG, EXT + EE + VEH, and EXT + NoEE + ORG did not differ significantly from the NoEXT control group (ps ≥ 0.34). In a similar fashion, PSD95 expression was significantly lower in EXT + NoEE + VEH group of male rats compared to the NoEXT control group (*p* = 0.02) and that groups receiving EXT + EE + ORG, EXT + EE + VEH, and EXT + NoEE + ORG did not differ significantly from the NoEXT control group (ps ≥ 0.17).

##### 3.1.4.2 Dorsal hippocampus

Among the male rats, there were no between-group differences in any of the nine protein targets in the analyses comparing the EXT + NoEE + VEH group to the Yoked Saline and NoEXT control groups and in the analyses comparing the four groups receiving EXT training (EXT + NoEE + VEH, EXT + EE + ORG, EXT + EE + VEH, and EXT + NoEE + ORG) to the NoEXT control group (Table 2).

##### 3.1.4.3 Ventromedial prefrontal cortex

As above, there were no between-group differences in any of the nine protein targets in the analyses comparing the EXT + NoEE + VEH group to the Yoked Saline and NoEXT control groups and in the analyses comparing the four groups of male rats receiving EXT training (EXT + NoEE + VEH, EXT + EE + ORG, EXT + EE + VEH, and EXT + NoEE + ORG) to the male NoEXT control group (Table 3).

##### 3.1.4.4 Nucleus accumbens

In analyses of male rats comparing the EXT + NoEE + VEH group to the Yoked Saline and NoEXT control groups, there were group differences in the relative expressions of pSer845GluA1 [*F*_(2, 21)_ = 4.6, *p* = 0.02], NR1 [*F*_(2, 21)_ = 7.7, *p* = 0.003; data transformed], total TrkB [*F*_(2, 21)_ = 9.0, *p* = 0.002; data transformed], and GABA_A_Rα1 [*F*_(2, 20)_ = 10.4, *p* = 0.001; data transformed]. As shown in Table 4, Dunnett *post-hoc* testing revealed that pSer845GluA1 (*p* = 0.02), NR1 (*p* = 0.002), total TrkB (*p* = 0.001), and GABA_A_Rα1 (*p* = 0.004) expressions were significantly lower in the EXT + NoEE + VEH group of male rats compared to the Yoked Saline control group. In the analyses to assess the effects of treatment in the four groups of male rats receiving EXT training (EXT + NoEE + VEH, EXT + EE + ORG, EXT + EE + VEH, and EXT + NoEE + ORG) relative to the NoEXT control group, there were no significant group differences in the relative expression for any of the 9 protein targets.

### 3.2 Experiment 2: effects of EXT + EE + ORG in female rats

#### 3.2.1 Cocaine baseline and extinction sessions

Cocaine self-administration behavior at baseline was similar in the two groups of female rats prior to initiating treatments. There were no significant group differences in the number of active [89.0 ± 26.1 vs. 153.4 ± 57.2; *t*_(10)_ = 1.03, *p* = 0.33] or inactive [30.3 ± 16.2 vs. 9.4 ± 4.7; *t*_(10)_ = 1.24, *p* = 0.24] lever responses, nor in the daily mg/kg cocaine intake [10.2 ± 2.0 vs. 12.7 ± 1.7; *t*_(10)_ = 0.96, *p* = 0.36] that were averaged over the last five sessions under the second-order schedule of cocaine delivery in the EXT + NoEE + VEH vs. EXT + EE + ORG groups, respectively. A separate 2-way ANOVA (group × sex) was conducted to determine if females responded differently than males to cocaine self-administration at baseline prior to initiating treatments. The EXT + NoEE + VEH and EXT + EE + ORG groups of each sex were compared. The results of this analysis showed greater cocaine self-administration behavior (active and inactive lever responses and daily mg/kg cocaine intake) at baseline in female than male rats (see Supplementary Figure 1).

**Figure 5:**
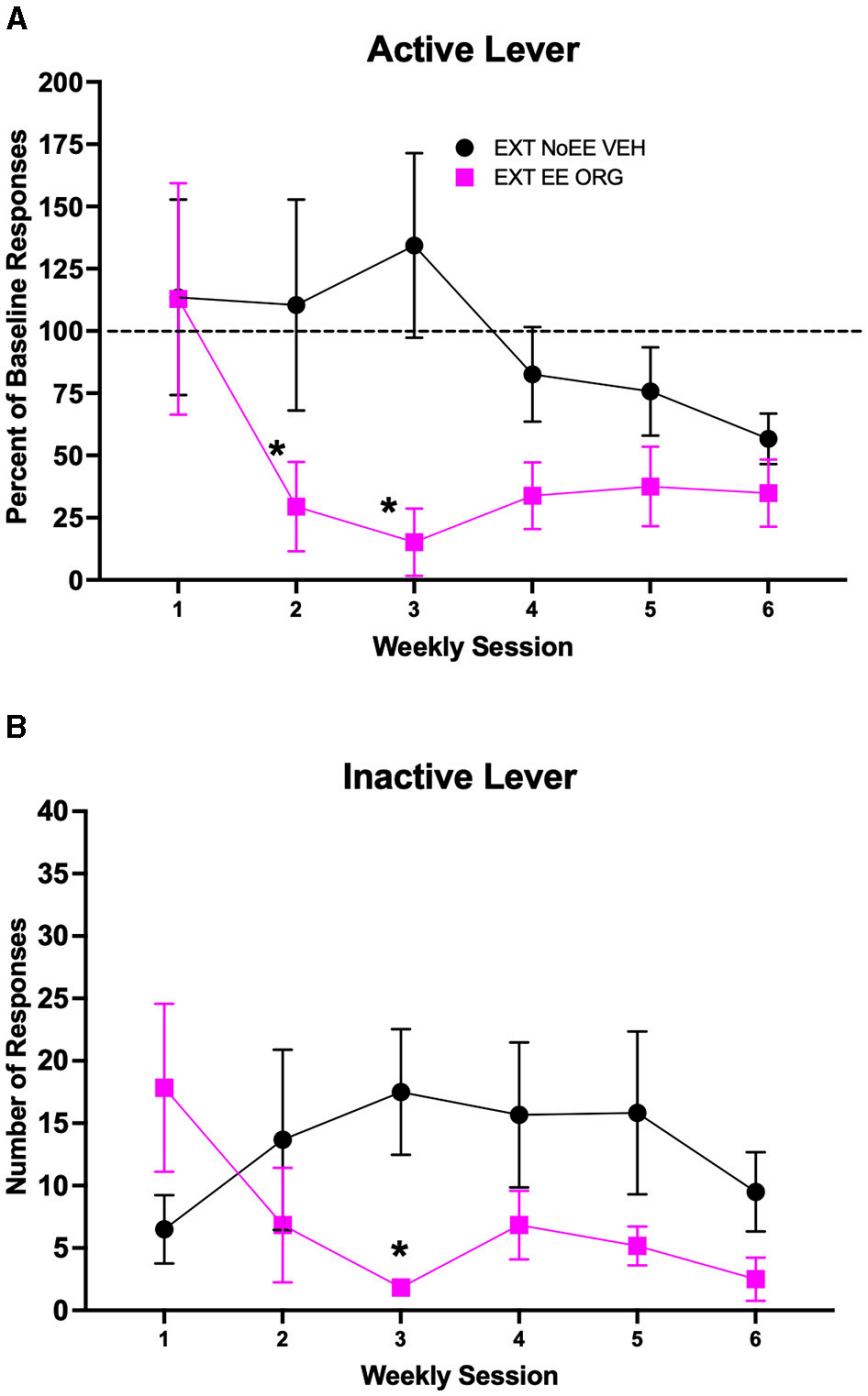
Extinction behavior in female rats. Groups consisted of rats who during the extinction phase received cocaine-cue extinction training with no-added treatment (EXT + NoEE + VEH; *n* =6) or with EE treatment combined with ORG (EXT + EE + ORG; *n* =6). Values are the mean ± s.e.m. for the percent of baseline active lever responses **(A)** and the number of inactive lever responses **(B)** for each of the 6 weekly extinction training sessions. *ps ≤ 0.044 compared to the EXT + NoEE + VEH group.

The EXT + EE + ORG treatment in female rats facilitated cocaine-cue extinction learning compared to extinction training alone (EXT + NoEE + VEH). Active lever responding (Figure 5A) declined across the course of extinction training [main effect of session number; *F*_(5, 50)_ = 3.02, *p* = 0.018], with significantly less responding during week 6 compared to week 1 overall (*p* ≤ 0.01). However, the magnitude of decline in active lever responding across the course of extinction training was different between the two groups of female rats [group × session number interaction; *F*_(5, 50)_ = 2.46, *p* = 0.045]. The group that received EXT + EE + ORG treatment responded significantly less than the group that received EXT + NoEE + VEH treatment during weeks 2–3 of extinction training (ps ≤ 0.044). Inactive lever responses (Figure 5B) remained at low levels in both groups across the course of extinction training, but there was a significant group X session number interaction [*F*_(5, 50)_ = 2.97, *p* = 0.02]. There was a significantly lower number of inactive lever responses in the EXT + EE + ORG treatment group compared to the EXT + NoEE + VEH treatment group during week 3 of extinction training (*p* = 0.02) but not during any other week of testing. A separate 3-way ANOVA (group × sex × session number) was conducted to determine if there were differential effects of EXT training and in response to EE + ORG treatment in female vs. male rats. The results of this analysis showed that EE + ORG treatment enhanced the rate of extinction learning similarly in male and female rats (see Supplementary Figure 2). Without EE + ORG treatment, extinction learning also was similar in male and female rats albeit at a slower rate compared to EE + ORG treatment.

#### 3.2.2 Molecular changes

##### 3.2.2.1 Basolateral amygdala

In analyses of female rats comparing the EXT + NoEE + VEH group (rats with cocaine and extinction histories only) and the EXT + EE + ORG group (rats with cocaine and extinction histories along with the multimodal treatment strategy) to the NoEXT control group, there were group differences in the relative expression of total GluA1 [*F*_(2, 29)_ = 19.3, *p* = 0.0001], GluA2 [*F*_(2, 29)_ = 4.3, *p* = 0.02], NR1 [*F*_(2, 29)_ = 6.5, *p* = 0.005], NR2B [*F*_(2, 29)_ = 3.3, *p* = 0.05; data transformed], total TrkB [*F*_(2, 29)_ = 5.9, *p* = 0.007], and PSD95 [*F*_(2, 29)_ = 33.9, *p* = 0.0001]. As shown in Table 1, Dunnett *post-hoc* testing revealed that total GluA1 (ps ≤ 0.0008), total TrkB (ps ≤ 0.049), and PSD95 (ps ≤ 0.0001) expressions were significantly lower in the EXT + NoEE + VEH and EXT + EE + ORG groups of female rats compared to the NoEXT control group. Only the EXT + EE + ORG group had significantly lower expression of GluA2 (*p* = 0.01), NR1 (*p* = 0.003), and NR2B (*p* = 0.047) compared to the NoEXT control group. The EXT + NoEE + VEH group did not differ significantly from the NoEXT control group for these latter protein targets (ps ≤ 0.12).

**Figure 6:**
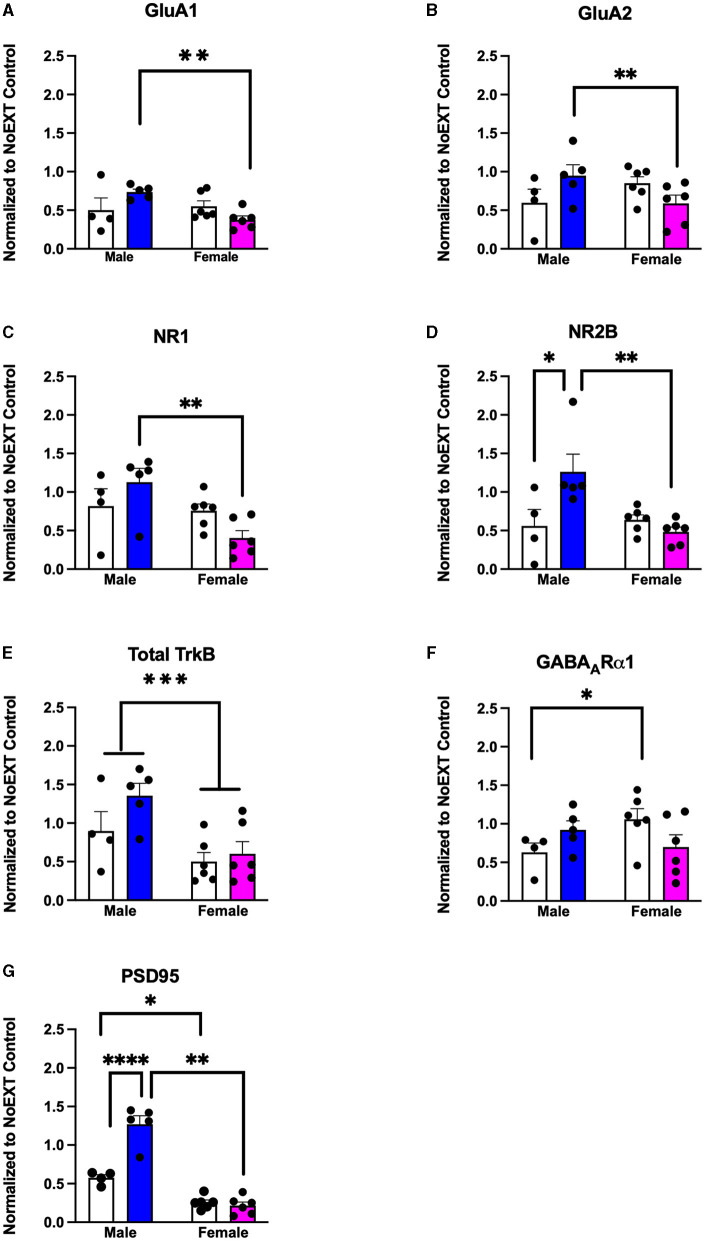
Sex differences in the expression levels of glutamate and non-glutamate receptor subunits in the basolateral amygdala. Groups consisted of male and female rats who during the extinction phase received cocaine-cue extinction training with no-added treatment (EXT + NoEE + VEH; *n* =4 males and *n* =6 females) or with EE treatment combined with ORG (EXT + EE + ORG; *n* = 5 males and *n* = 6 females). Tissue samples were probed via western blot analysis for expression of AMPAR subunits GluA1 **(A)** and GluA2) **(B)**, NMDAR subunits NR1 **(C)** and NR2B **(D)**, Total TrkB **(E)**, GABA_A_Rα1 **(F)**, and PSD95 **(G)**. Values are individual data points and the mean ± s.e.m. normalized to the NoEXT control. *ps ≤ 0.05, **ps ≤ 0.05, ***p =0.003, ****p = 0.0001.

In analyses to determine sex differences in protein expression in the BLA (Figure 6), male and female rats receiving EXT + NoEE + VEH and EXT + EE + ORG were compared. There were main effects of sex for total TrkB [*F*_(1, 17)_ = 11.9, *p* = 0.003], with female rats overall expressing significantly lower levels than male rats. Significant sex × treatment interactions were revealed for total GluA1 [*F*_(1, 17)_ = 6.7, *p* = 0.02], GluA2 [*F*_(1, 17)_ = 6.1, *p* = 0.02), NR1 [*F*_(1, 17)_ = 5.4, *p* = 0.03], NR2B [*F*_(1, 17)_ = 8.7, *p* = 0.009], GABA_A_Rα1 [*F*_(1, 17)_ = 5.3, *p* = 0.03], and PSD95 [*F*_(1, 17)_ = 32, *p* = 0.0001]. Tukey *post-hoc* testing revealed that for groups receiving the EXT + EE + ORG treatment, female rats had lower expression levels of GluA1 (*p* = 0.02), GluA2 (*p* = 0.05), NR1 (*p* = 0.002), NR2B (*p* = 0.001), and PSD95 (*p* = 0.0001) than male rats. For groups receiving the EXT + NoEE + VEH treatment, female rats had greater expression levels of GABA_A_Rα1 (*p* = 0.05) and lower expression levels of PSD95 (*p* = 0.003) than male rats. Notably, male rats receiving EXT + EE + ORG treatment had greater expression levels of NR2B (*p* = 0.006) and PSD95 (*p* = 0.0001) than male rats receiving EXT + NoEE + VEH treatment. This positive influence of EXT + EE + ORG treatment on NR2B and PSD95 in male rats was not observed in female rats.

##### 3.2.2.2 Dorsal hippocampus

There were group differences in the relative expression of total GluA1 [*F*_(2, 29)_ = 7.7, *p* = 0.002; data transformed], pSer845GluA1 [*F*_(2, 29)_ = 21.3, *p* = 0.0001], NR1 [*F*_(2, 29)_ = 4.2, *p* = 0.02], total TrkB [*F*_(2, 29)_ = 8.1, *p* = 0.002; data transformed], GABA_A_Rα1 [*F*_(2, 29)_ = 38.0, *p* = 0.0001; data transformed], and PSD95 [F_(2, 29)_ = 5.8, *p* = 0.008]. As shown in Table 2, Dunnett *post-hoc* testing revealed that compared to the NoEXT control, the expression of total GluA1 (*p* = 0.001) and total TrkB (*p* = 0.005) was significantly greater in the EXT + NoEE + VEH group and that the expression of NR1 (*p* = 0.02) and PSD95 (*p* = 0.004) was significantly lower in the EXT + EE + ORG group. Both groups of female rats had significant lower expression of pSer845GluA1 compared to the NoEXT control (ps ≤ 0.0005), Notably, both groups of female rats had significantly greater expression of GABA_A_Rα1 compared to the NoEXT control (ps ≤ 0.005).

**Figure 7:**
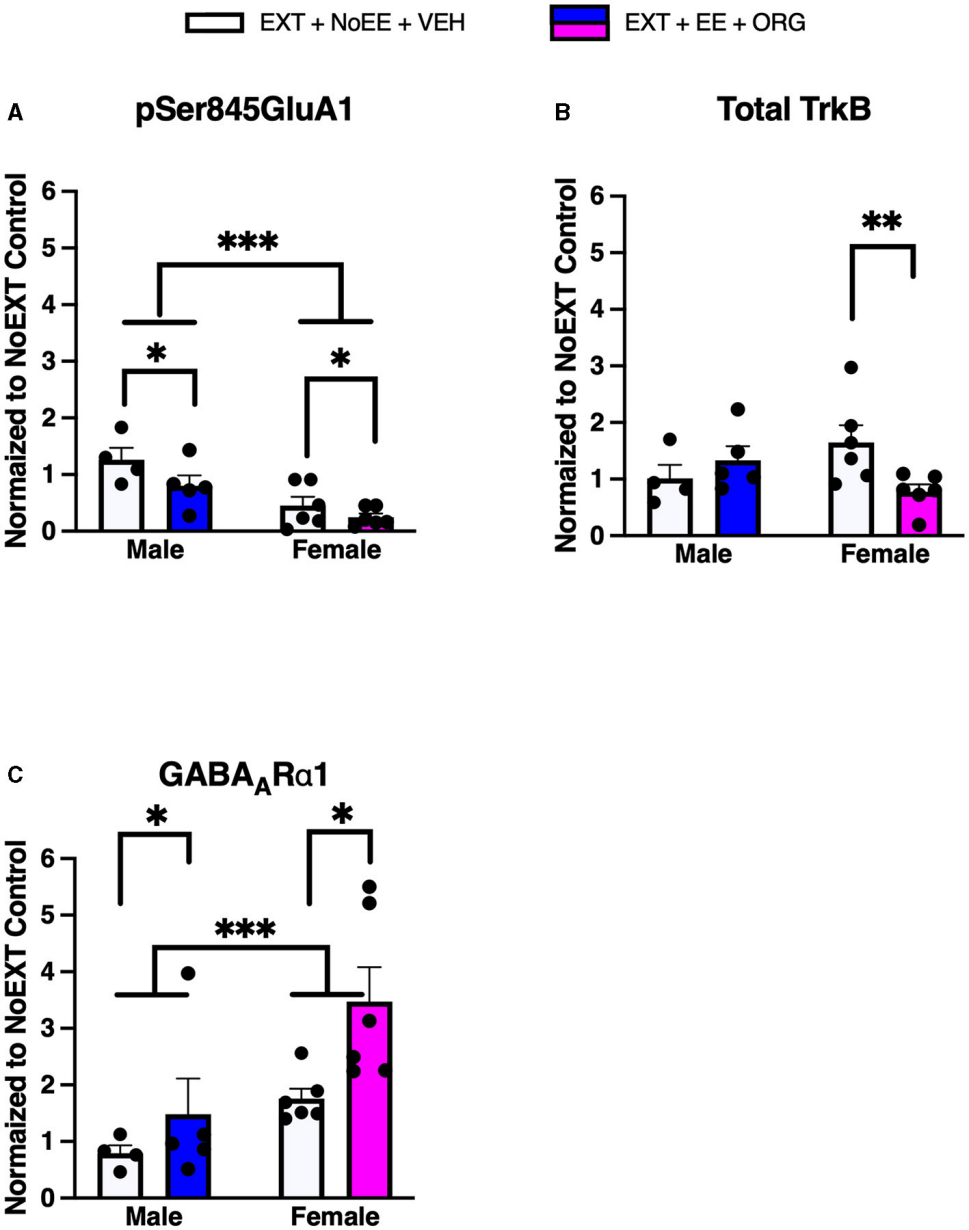
Sex differences in the expression levels of glutamate and non-glutamate receptor subunits in the dorsal hippocampus. Groups consisted of male and female rats who during the extinction phase received cocaine-cue extinction training with no-added treatment (EXT + NoEE + VEH; *n* = 4 males and *n* = 6 females) or with EE treatment combined with ORG (EXT + EE + ORG; *n* = 5 males and *n* = 6 females). Tissue samples were probed via western blot analysis for expression of pSer845GluA1 **(A)**, Total TrkB **(B)**, and GABA_A_Rα1 **(C)**. Values are individual data points and the mean ± s.e.m. normalized to the NoEXT control. *ps ≤ 0.05, **p = 0.015, ***ps ≤ 0.007.

**Figure 8:**
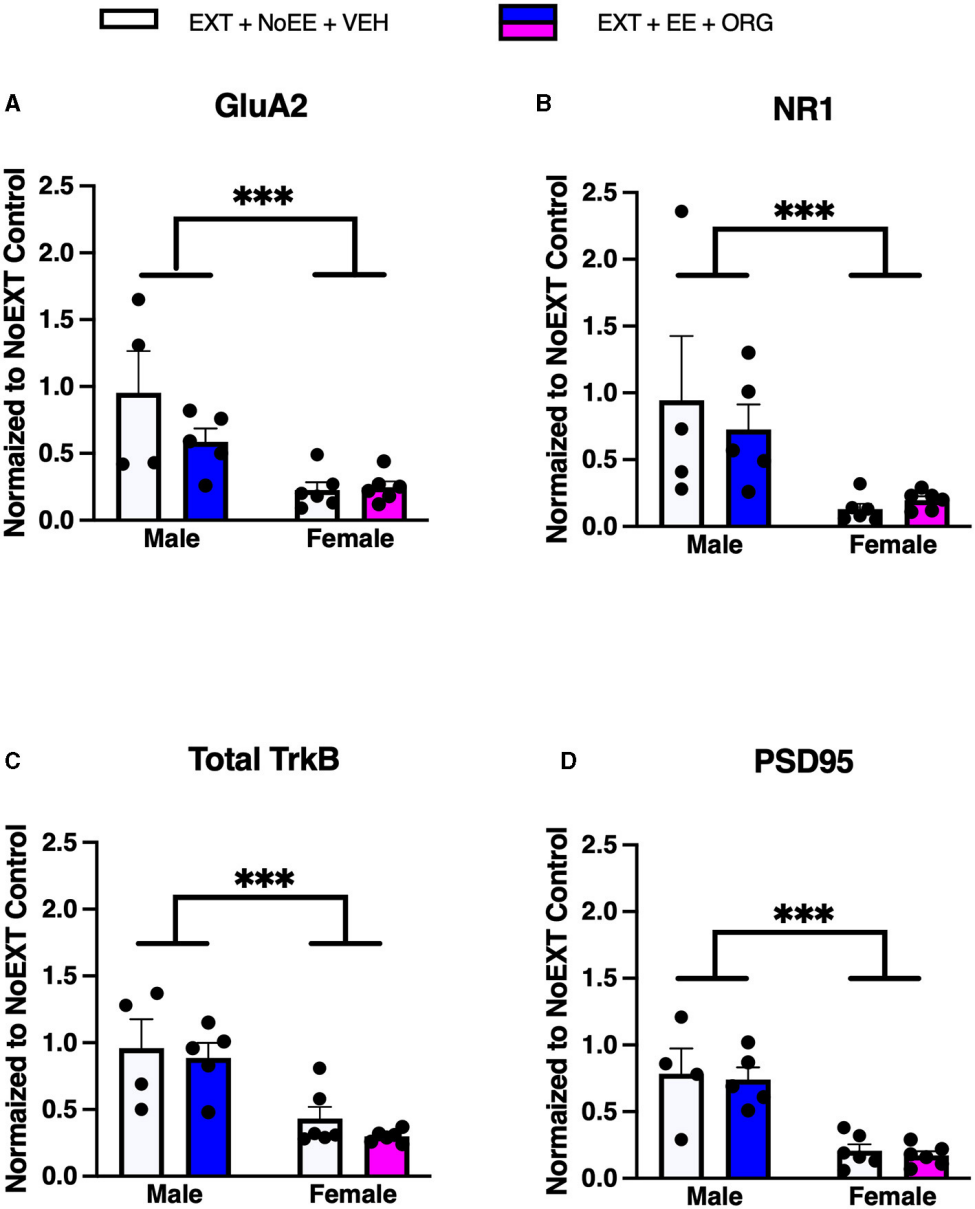
Sex differences in the expression levels of glutamate and non-glutamate receptor subunits in the ventromedial prefrontal cortex. Groups consisted of male and female rats who during the extinction phase received cocaine-cue extinction training with no-added treatment (EXT + NoEE + VEH; *n* = 4 males and *n* = 6 females) or with EE treatment combined with ORG (EXT + EE + ORG; *n* = 5 males and *n* = 6 females). Tissue samples were probed via western blot analysis for expression of GluA2 **(A)**, NR1 **(B)**, Total TrkB **(C)**, and PSD95 **(D)**. Values are individual data points and the mean ± s.e.m. normalized to the NoEXT control. ***ps ≤ 0.004.

In analyses to determine sex differences in protein expression in the DH, male and female rats receiving EXT + NoEE + VEH and EXT + EE + ORG were compared. There were main effects of sex [*F*_(1, 17)_ = 19.5, *p* = 0.0004] and treatment [*F*_(1, 17)_ = 4.6, *p* = 0.047] for pSer845GluA1 as well as main effects of sex [*F*_(1, 17)_ = 9.3, *p* = 0.007] and treatment [*F*_(1, 17)_ = 6.2, *p* = 0.02] for GABA_A_Rα1. Tukey *post-hoc* testing revealed that female rats expressed significantly lower levels of pSer845GluA1 (*p* = 0.0004) and significantly greater levels of GABA_A_Rα1 (*p* = 0.007) than male rats overall (Figure 7). For the treatment main effects, expression levels were significantly lower overall after the EXT + EE + ORG treatment compared to the EXT + NoEE + VEH treatment for pSer845GluA1 (*p* = 0.047) and were significantly greater overall after the EXT + EE + ORG treatment compared to the EXT + NoEE + VEH treatment for GABA_A_Rα1 expression (*p* = 0.02). For total TrkB, there was a sex X treatment interaction [*F*_(1, 17)_ = 5.9, *p* = 0.03]. Notably, Tukey *post-hoc* testing revealed that there were lower levels of total TrkB expressed after the EXT + EE + ORG treatment compared to the EXT + NoEE + VEH treatment in female (*p* = 0.015) but not male rats (Figure 8).

##### 3.2.2.3 Ventromedial prefrontal cortex

There were group differences in the relative expression of GluA2 [*F*_(2, 29)_ = 30.2, *p* = 0.0001; data transformed], NR1 [*F*_(2, 29)_ = 21.7, *p* = 0.0001; data transformed], NR2B [*F*_(2, 29)_ = 4.6, *p* = 0.0]), total TrkB [*F*_(2, 29)_ = 22.1, *p* = 0.001], and PSD95 [*F*_(2, 29)_ = 22.6, p = 0.0001; data transformed] in the vmPFC. As shown in Table 3, Dunnett *post-hoc* testing revealed that the expression of GluA2 (ps ≤ 0.0001), NR1 (ps ≤ 0.0001), NR2B (ps ≤ 0.05), total TrkB (ps ≤ 0.0002), and PSD95 (ps ≤ 0.0001) was significantly lower in the EXT + NoEE + VEH and EXT + EE + ORG groups compared to the NoEXT control.

In analyses to determine sex differences in protein expression in the vmPFC, male and female rats receiving EXT + NoEE + VEH and EXT + EE + ORG were compared. There were main effects of sex for GluA2 [*F*_(1, 17)_ = 16.1, *p* = 0.0009], NR1 [*F*_(1, 17)_ = 11.0, *p* = 0.004], Total TrkB [*F*_(1, 17)_ = 25.5, *p* = 0.0001], and PSD95 [*F*_(1, 17)_ = 40.6, *p* = 0.0001]. Tukey *post-hoc* testing revealed that female rats expressed significantly (ps ≤ 0.004) lower levels of each of these four protein targets than male rats (Figure 8).

##### 3.2.2.4 Nucleus accumbens

There were group differences in the relative expression of total GluA1 [*F*_(2, 24)_ = 3.8, *p* = 0.037; data transformed], GluA2 [*F*_(2, 24)_ = 15.4, *p* = 0.0001], NR1 [*F*_(2, 25)_ = 63.8, *p* = 0.00001; data transformed], GABA_A_Rα1 [*F*_(2, 24)_ = 60.6, *p* = 0.0001; data transformed], and PSD95 [*F*_(2, 25)_ = 3.6, *p* = 0.044] in the NAc. As shown in Table 4, Dunnett *post-hoc* testing revealed that the expression of GluA2 was significantly lower (ps ≤ 0.004) and the expression of NR1 (ps ≤ 0.0001) and GABA_A_Rα1 (ps ≤ 0.0001) was significantly greater in the EXT + NoEE + VEH and EXT + EE + ORG groups compared to the NoEXT control. The expression of total GluA1 and PSD95 was different only in the EXT + EE + ORG group, with significantly greater expression of total GluA1 (*p* = 0.034) and significantly lower expression of PSD95 (*p* = 0.026) compared to the NoEXT control.

**Figure 9:**
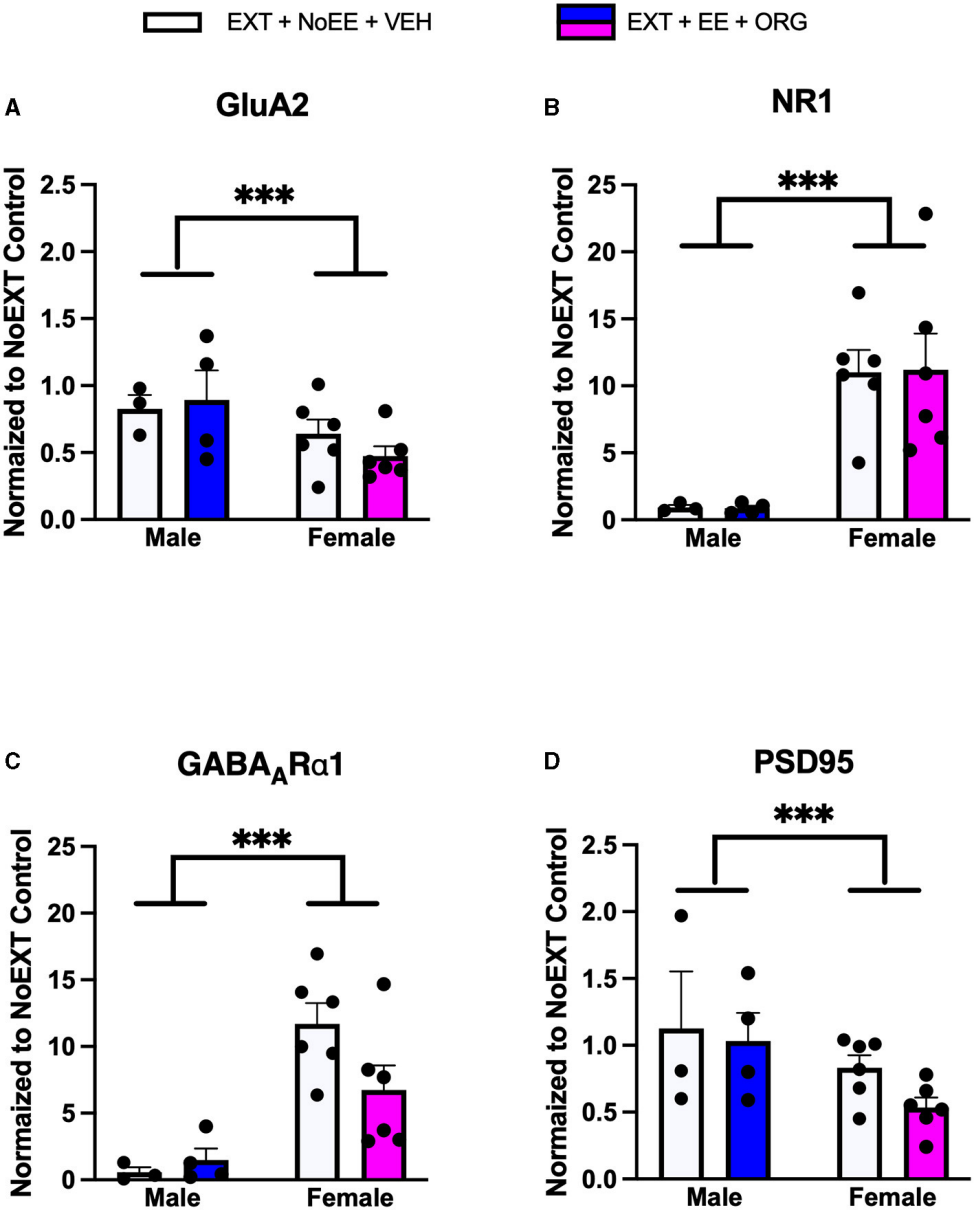
Sex differences in the expression levels of glutamate and non-glutamate receptor subunits in the nucleus accumbens. Groups consisted of male and female rats who during the extinction phase received cocaine-cue extinction training with no-added treatment (EXT + NoEE + VEH; *n* = 3 males and *n* = 6 females) or with EE treatment combined with ORG (EXT + EE + ORG; *n* = 4 males and *n* = 6 females). Tissue samples were probed via western blot analysis for expression of GluA2 **(A)**, NR1 **(B)**, GABA_A_Rα1 **(C)**, and PSD95 **(D)**. Values are individual data points and the mean ± s.e.m. normalized to the NoEXT control. ***ps ≤ 0.04.

In analyses to determine sex differences in protein expression in the NAc, male and female rats receiving EXT + NoEE + VEH and EXT + EE + ORG were compared. There were main effects of sex for GluA2 [*F*_(1, 15)_ = 5.2, *p* = 0.034], NR1 [*F*_(1, 15)_ = 22.5, *p* = 0.0003], GABA_A_Rα1 [*F*_(1, 15)_ = 23.5, *p* = 0.0002], and PSD95 [*F*_(1, 15)_ = 5.0, *p* = 0.041]. Tukey *post-hoc* testing revealed that female rats expressed significantly lower levels of GluA2 (ps ≤ 0.034) and PSD95 (*p* = 0.041) and significantly greater levels of NR1 (*p* = 0.0003 and GABA_A_Rα1 (*p* = 0.0002) than male rats overall (Figure 9).

## 4 Discussion

The behavioral observations in the current study extend our earlier report (Kantak et al., 2020) by showing that the beneficial effects of EE + ORG for cocaine relapse prevention in male rats trained to self-administer a high 1.0 mg/kg training dose of cocaine required combining these treatment interventions with cocaine-cue extinction training. The results of these and other earlier investigations that utilized a more moderate 0.3 mg/kg training dose of cocaine (Achat-Mendes et al., 2012; Gauthier et al., 2017) collectively suggest that combining both EE and ORG with cocaine-cue extinction training is optimal for reducing relapse to cocaine self-administration across low and high baseline levels of cocaine use, at least in male rats, as EE + ORG was the only treatment that consistently inhibited reacquisition of 1.0 mg/kg cocaine self-administration. Of importance, treatment with EE + ORG facilitated the rate of cocaine-cue extinction learning in both male and female rats (Kantak et al., 2020; present findings), however, this outcome did not result in a reduction in the reacquisition of 1.0 mg/kg cocaine self-administration in the females (Kantak et al., 2020). These results illustrate clear sex differences in the efficacy of this multimodal treatment intervention for producing a lasting enhancement of extinction memory for cocaine relapse prevention after reexposure to cocaine. As sex differences in treatment efficacy are crucial for therapeutic design, we expanded our earlier findings to understand the molecular differences underlying (1) the effects of EE + ORG in male rats and (2) the lack of efficacy of EE + ORG in female rats. To this end, we compared the protein expression levels of male rats without extinction training (NoEXT) to those with extinction training with or without EE + ORG, allowing us to elucidate any EE + ORG-specific effects on protein expression. To study sex differences, we compared protein expression across extinction-trained male and female rats with or without EE + ORG, allowing us to observe if and how EE + ORG differentially affects protein expression after extinction training based on sex.

### 4.1 Molecular differences between treatments

GlyT-1 inhibitors, such as ORG, indirectly activate NMDA receptors (NMDARs) through an increase in synaptic glycine (Harsing et al., 2012), a co-agonist of NMDARs (Thomson et al., 1989; Thomson, 1990). Glycine binding on NMDARs has been implicated in the induction of long-term potentiation (LTP) as well as short-term plasticity (Bashir et al., 1990; Lu et al., 2001; Krasteniakov et al., 2005; Zhang et al., 2006; Igartua et al., 2007; Chen et al., 2011). Accordingly, glycine has significant effects on the trafficking, synaptic retention, and activation of NMDARs and AMPA receptors (AMPARs) (Thomson et al., 1989; Shahi and Baudry, 1993; Chen et al., 2011; Zhang et al., 2014). To improve learning and memory, AMPARs must undergo phosphorylation at serine residue 845 on the GluA1 subunit (pSer845GluA1), translocate to synaptic surfaces, and anchor to synaptic sites via association with receptor interacting proteins (Malinow and Malenka, 2002; Lee et al., 2003; Man et al., 2007; Li and Wolf, 2011). Activation of NMDARs and TrkB influence these AMPAR-related processes. EE on the other hand enhances expression of the NR2B subunit of NMDARs (Tang et al., 2001) and it also indirectly activates TrkB through the release of BDNF (Segovia et al., 2006; Bechara et al., 2014). Consequently, site-specific changes in NR1, NR2B, GluA1, and TrkB protein expression may be particularly relevant for understanding the interactive effects of EE + ORG with extinction training for cocaine relapse prevention. In male rats, we found that there were no protein expression changes between the NoEXT and EXT + NoEE + VEH groups in the ventromedial prefrontal cortex (vmPFC) or dorsal hippocampus (DH), so we believe that the effects of EXT training in males are primarily due to molecular modulation in the NAc and BLA. In these regions, we found that male rats with cocaine-cue extinction training (EXT + NoEE + VEH group) exhibited significant reductions in the expression of several protein targets compared to the yoked-saline control group. Unexpectedly, male rats with EXT + EE + ORG had a reversal of these decreases and the difference in protein expression was statistically insignificant compared to the NoEXT control group. This is interesting as it may suggest that changes in overall protein expression seem not to be driving the effects of EXT + EE + ORG on cocaine self-administration behavior. It is possible that the behavioral effects caused by EXT + EE + ORG are due to activation of different signaling pathways, changes in protein modifications, and/or different receptor subcellular distribution and synaptic activity.

In the NAc, several proteins (pSer845GluA1, NR1, total TrkB, and GABA_A_Rα1) were decreased in the EXT + NoEE + VEH group compared to the YS control group. Expression of these proteins was rescued in the EXT + EE + ORG group, back to levels that are comparable to the NoEXT groups. Since there were no significant changes in protein expression between the NoEXT and EXT + EE + ORG groups, we hypothesize that the behavioral effects of EE + ORG are mediated by altered plasticity, mediated by changes in NMDAR and AMPAR activation. It has been shown that cocaine exposure impairs AMPAR-dependent plasticity in the NAc (Wolf and Ferrario, 2010) and inhibits long term depression (LTD) in the NAc (Martin et al., 2006). LTD is a form of Hebbian plasticity that is mediated by AMPARs and NMDARs and contributes to neuronal circuit remodeling, memory consolidation or erasure, and reward-seeking behavior in the NAc (Braunewell and Manahan-Vaughan, 2001; Massey and Bashir, 2007; Collingridge et al., 2010; Luscher and Malenka, 2012; Vega-Villar et al., 2019). Our hypothesized restoration of plasticity by EE + ORG is supported by previous findings regarding synaptic proteins in the NAc. Ours and others previous findings have found that EE increases TrkB and BDNF signaling in other brain regions (Hastings et al., 2020; Rojas-Carvajal et al., 2020). In addition, it has been shown that enhanced TrkB activity in the NAc strengthens cocaine extinction, specifically through modulation of NMDARs, and TrkB antagonism reduces self-administration (Otis et al., 2014; Verheij et al., 2016). Our finding that pSer845GluA1 expression is rescued is also consistent with our plasticity hypothesis. Phosphorylation at serine 845 increases synaptic expression of GluA1: pS845GluA1 has decreased internalization, resulting in increased synaptic retention (Man et al., 2007; Sathler et al., 2021). EE + ORG treatment restoring basal expression of pSer845GluA1 would also restore synaptic expression of GluA1, even if total GluA1 remains unchanged. In addition, expression of pSer845GluA1 has been shown to lower the threshold for induction of long term plasticity (Makino et al., 2011). In regard to our finding that GABA_A_Rα1 expression is decreased by EXT, previous studies have found that increased glutamatergic signaling results in increased extracellular GABA in the NAc, and increased GABA blocks cocaine self-administration (Kushner et al., 1999; Segovia and Mora, 2005), suggesting an important role for GABAergic signaling in cocaine addiction. Future studies could test this hypothesis by measuring synaptic protein expression, as opposed to total protein expression, or by using electrophysiological recording to measure changes in neuronal firing.

In the BLA, EXT led to a significant reduction of key proteins (total GluA1, NR1, NR2B, and PSD95) and EXT + EE + ORG rescued these effects. We have previously found similar decreases in GluA1 in the BLA after cocaine-cue extinction training, as compared to the YS control group (Nic Dhonnchadha et al., 2013). In agreement, it has previously been shown that glycine agonism enhances GluA1 synaptic insertion (Lu et al., 2001; Shelkar et al., 2021). One interesting finding in the BLA was restoration of expression of PSD95. PSD95 is a scaffolding protein that forms complexes with NMDARs and indirectly with AMPARs to regulate receptor localization and plasticity (Sheng, 2001; Béïque and Andrade, 2003; Béïque et al., 2006; Delint-Ramirez et al., 2010). In the BLA, PSD95 contributes to fear learning and avoidance behavior by coupling with neuronal nitric oxide (nNOS), which is produced following NMDAR activation (Cai et al., 2018; Li et al., 2018). This may suggest that alterations in PSD95 contribute to accumulation of AMPARs and NMDARs at the synapse, increasing synaptic activity. This proposed pathway agrees with our findings in female rats showing that expression of PSD95 remained significantly decreased after EE + ORG treatment. This outcome in female rats might also contribute to why the EE + ORG treatment intervention did not inhibit reacquisition of 1 mg/kg cocaine self-administration in the females (Kantak et al., 2020).

### 4.2 Molecular differences between sexes

We found numerous sex differences in protein expression that may contribute to the male-biased efficacy of EE + ORG for cocaine relapse prevention (Kantak et al., 2020). While there were brain region-specific changes, we found that generally female rats had lower expression of proteins related to excitatory signaling (such as AMPAR and NMDAR subunits) and higher expression of proteins related to inhibitory signaling (GABA_A_Rα1) after extinction training, as compared to their male counterparts. This suggests a sex-specific effect of extinction training on neuroplasticity may be one factor that contributes to the inability of EE + ORG to inhibit reacquisition of cocaine self-administration in female rats.

In the BLA, male rats that had received EE + ORG treatment during cocaine-cue extinction training had higher levels of glutamate receptor subunits (GluA1, GluA2, NR1, and NR2B) than female rats that received the same treatment. Trafficking and synaptic expression of these receptors are directly coupled to neuroplasticity (Thomson et al., 1989; Shahi and Baudry, 1993; Chen et al., 2011; Zhang et al., 2014). While not much is known about sexual dimorphisms in BLA neuroplasticity, there are findings that female rats have decreased dendritic spine density in the BLA, as compared to males (Rubinow et al., 2009).

Female rats also had reduced expression of total TrkB in the DH and vmPFC after EXT + EE + ORG, as compared to extinction training alone. Low expression levels of total TrkB across brain regions after EE + ORG treatment in female rats may be a second factor contributing to the inability of EE + ORG to inhibit reacquisition of cocaine self-administration in the females, as TrkB and BDNF signaling are crucial for neuroplasticity (Musumeci et al., 2009; Yoshii and Constantine-Paton, 2010).

Interestingly, we found that after extinction training, regardless of EE + ORG treatment, females had significantly lower expression of pS845GluA1 in the DH and higher expression of GABA_A_Rα1 in the DH and NAc. It has been shown that EE increases levels of glutamate and NMDARs in male rats but decreases glutamate levels in female rats (Kokras et al., 2019). In other models of addiction, increased NAc GABAergic transmission is associated with increased aversive effects of withdrawal (Hwang et al., 1990; Carcoba et al., 2018). These results highlight a sex-dependent change in NAc GABAergic transmission associated with use of addictive substances, and that this female-specific increase leads to more severe withdrawal and relapse. Based on this, it is possible that males are more sensitive to EE than females due to increased neuroplasticity or propensity to favor excitatory signaling.

The sex differences could result from changes in neural projection and neurocircuitry activities. We found that female rats had lower expression of GluA2, NR1 and PSD95 after extinction training, regardless of EE + ORG treatment. It has been shown that inhibition of vmPFC neurons prevents the ability of extinction training to inhibit cue-associated cocaine preference (Van Den Oever et al., 2008, 2013; Miller et al., 2017). It has also been shown that projections from the vmPFC to the NAc are necessary to inhibit drug seeking after extinction (Peters et al., 2009). Based on this, the decreased expression of excitatory synaptic proteins in the vmPFC of female rats, as compared to males, may result in decreased input from the vmPFC to the NAc and contribute to the inability of EE + ORG to inhibit reacquisition of cocaine self-administration in the females.

The sex-dependent changes in neuroplasticity after extinction training could be attributable to differences in sex hormone signaling in these key brain areas. Notably, estrogen has been shown to increase the propensity for reinstatement of extinguished cocaine-seeking behavior (Larson and Carroll, 2007) and removal of endogenous estrogen by ovariectomy decreases cocaine-primed reinstatement of extinguished cocaine-seeking behavior (Larson et al., 2005). In addition, it has been shown that females self-administer cocaine more during the estrus phase of the estrous cycle, suggesting that estrogen affects reward and motivation (Kerstetter et al., 2008). Estrogen signaling has significant effects on neuronal structure, dendritic arborization, and synaptic plasticity (McEwen, 1999; Hyer et al., 2018; Gall et al., 2023) and it has been shown that proper estrogen receptor expression is necessary for the excitation/inhibition (E/I) balance (Wang et al., 2021). Interestingly, the effects of testosterone on enhancing cocaine self-administration also rely on the activation of estrogen receptors (Menéndez-Delmestre et al., 2024). Thus, future studies could explore the implication of estrogen signaling in the molecular cascade underlying cocaine extinction in females.

### 4.3 Conclusions

Male rats were more sensitive than female rats to the neuroplasticity-inducing effects of EE + ORG. This treatment prevented reductions in total GluA1 and PSD95 protein expression selectively in the BLA of male rats trained to self-administer cocaine and receiving cocaine-cue extinction training. These treatment-associated changes could be critically important for the ability of EE + ORG to improve extinction memory retrieval for inhibiting cocaine relapse. Female rats were deficient in the expression levels of multiple protein targets across the 4 brain regions, especially after EE + ORG treatment. These include total GluA1 and PSD95 in the BLA, as well as total TrkB in the BLA, DH, and vmPFC. Interestingly, the procognitive effects of genetic deletion of the GlyT-1 transporter (a functional proxy for GlyT-1 inhibition) on extinction memory shows sex differences, with mutant male mice exhibiting enhanced extinction to an appetitive conditioned stimulus and mutant female mice being resistant to such extinction training (Dubroqua et al., 2011). Sex differences in brain chemistry and behavior after EE exposure also have been reported in rodents. For example, EE increases expression of BDNF in hippocampus and frontal cortex (Chourbaji et al., 2012; Vinogradova et al., 2023) and expression of NR2B in hippocampus (Kokras et al., 2019) in males but not females. EE also has memory-enhancing effects in males but not females (Sakhaie et al., 2020). Of additional relevance to the present investigation are finding showing that administration of a TrkB agonist enhanced cued fear extinction in male but not female mice (Tohyama et al., 2020). Thus, it is not surprising from a mechanistic perspective that the EE + ORG treatment intervention with cocaine-cue extinction training was effective in male rats but not in female rats for inhibiting relapse to cocaine self-administration.

The results of these studies may provide new insights for the development of alternative strategies that can induce in female subjects the kinds of neuroplasticity needed for cocaine relapse prevention. Clinical research supports this need. For example, during cue-exposure therapy in individuals with a cocaine use disorder, men were better able than women to reduce their arousal to cocaine stimuli and exert better control over reactivity to cocaine cue presentation (Sterling et al., 2004). Others have shown that in individuals with cocaine and other substance use disorders, cue exposure caused greater anxiety, craving, and drug use days at follow-up in women as compared to men (Smith et al., 2023). The present investigation supports the clinical view that differential pharmacological and behavioral treatment approaches may be needed during cue exposure therapy in men vs. women to inhibit relapse to the use of cocaine and other drugs. Our animal model of cue exposure therapy could serve as a translational bridge to help establish the efficacy of novel extinction-based treatments in males vs. females. Future studies are needed to validate the hypothesis that the molecular changes lead to regulation in synaptic plasticity which contributes to the effects of EE + ORG. In addition, to verify if increases in total GluA1 in the BLA and total TrkB in the BLA, DH, or vmPFC are causal for the cocaine relapse-inhibiting effects of EE + ORG treatment, virus brain injection could be used to determine if overexpression of these protein targets during extinction training can mimic the effects of EE + ORG and if silencing these protein targets during extinction training can block the effects of EE + ORG.

As a final note, it is important to point out that drug-seeking responses during the cocaine self-administration reacquisition phase in male rats was reduced to approximately 60% of baseline and that cocaine intake was reduced to approximately 50% of baseline for a prolonged period after receiving the EE + ORG treatment during cocaine-cue extinction training. While complete cocaine abstinence was not reached, the effects of EE + ORG may be best described as a means to achieve harm reduction. Non-abstinence endpoints for cocaine use disorder are gaining traction in the clinical literature and such efforts support harm reduction as a clinical endpoint (Roos et al., 2019). Currently, relapse is a common outcome for substance use disorder treatment: Studies have shown that only 60% of patients stay abstinent after just 3 months, and only 50% of patients remain abstinent after just 1 year (McKay et al., 2013). It has been shown that drug users are less likely to relapse when provided with more supports such as concurrent psychological treatment or medication-assisted treatments (Van Der Woerd et al., 2010; Gallagher et al., 2019). Our present findings shed an important light on how these co-treatments (such as EE + ORG) can facilitate harm reduction by affecting neuroplasticity. Moreover, our general EXT + EE + ORG model can be manipulated to study the effects of combinatorial treatment—such as paired psychological and pharmacological intervention—on promoting long-term reductions in drug use.

## Data availability statement

The raw data supporting the conclusions of this article will be made available by the authors, without undue reservation.

## Ethics statement

The animal study was approved by Institutional Care and Usage Committee (IACUC), Boston University Charles River Campus. The study was conducted in accordance with the local legislation and institutional requirements.

## Author contributions

LP: Data curation, Formal analysis, Investigation, Validation, Writing – original draft, Writing – review & editing. JN: Data curation, Investigation, Writing – review & editing. NG: Formal analysis, Investigation, Writing – review & editing. PR-E: Data curation, Investigation, Writing – review & editing. HQ: Writing – review & editing, Data curation, Formal analysis, Investigation, Validation. SG: Data curation, Investigation, Writing – review & editing. H-YM: Conceptualization, Funding acquisition, Methodology, Project administration, Supervision, Writing – original draft, Writing – review & editing. KK: Conceptualization, Funding acquisition, Methodology, Project administration, Supervision, Visualization, Writing – original draft, Writing – review & editing.
