# Supplementary material for: Molecular mechanisms underlying sex and treatment-dependent differences in an animal model of cue-exposure therapy for cocaine relapse prevention
Source: Front Neurosci. 2024 Aug 8;18:1425447. doi: 10.3389/fnins.2024.1425447 (PMC11339646; doi:10.3389/fnins.2024.1425447)
Supplement: Supplementary file 1 [file Data_Sheet_1.docx]

**Supplemental Results**

**No Extinction Control Data in Male Rats**

Because there were no significant differences in active and inactive lever responding and daily cocaine intake between the four NoEXT treatment groups across the last 5 baseline sessions (Supplemental Table 1) and across the 15 reacquisition sessions (Supplemental Table 2), these four groups of rats were combined to form a single NoEXT control group to compare against the four EXT treatment groups in the main analyses.

**Supplemental Table 1. Self-Administration Behavior at Baseline in the Four NoEXT Treatment Groups**

| Treatment Group | Day 1 | Day 2 | Day 3 | Day 4 | Day 5 | Avg. Days 1-5 |
| --- | --- | --- | --- | --- | --- | --- |
|  |  |  |  |  |  |  |
| NoEXT | 60±7 | 61±9 | 69±9 | 66±9 | 75±16 | 66±9 |
| NoEXT+EE | 63±10 | 69±14 | 60±10 | 64±11 | 61±12 | 63±11 |
| NoEXT+ORG | 59±13 | 81±35 | 55±11 | 52±9 | 53±8 | 60±14 |
| NoEXT+EE+ORG | 59±13 | 63±9 | 57±9 | 61±8 | 65±8 | 61±8 |

Active Lever Responses

**Source of Variation DF SS MS F P**

Group x Session Number 12 6411 534.2 0.940 0.51

Session Number 4 2197 549.1 0.966 (df 1.71, 63.3) 0.37

Group 3 1289 429.6 0.069 0.98

Subject 37 230005 6216.0

Residual 148 84105 568.3

Inactive Lever Responses

| Treatment Group | Day 1 | Day 2 | Day 3 | Day 4 | Day 5 | Avg. Days 1-5 |
| --- | --- | --- | --- | --- | --- | --- |
|  |  |  |  |  |  |  |
| NoEXT | 4±2 | 4±1 | 4±1 | 2±1 | 3±1 | 3±1 |
| NoEXT+EE | 4±1 | 7±3 | 3±1 | 4±1 | 2±1 | 4±1 |
| NoEXT+ORG | 5±2 | 5±2 | 4±2 | 4±2 | 3±2 | 4±2 |
| NoEXT+EE+ORG | 6±3 | 5±2 | 5±3 | 4±1 | 5±1 | 5±2 |

**Source of Variation DF SS MS F P**

Group x Session Number 12 110.3 9.2 0.730 0.72

Session Number 4 112.7 28.2 2.236 (df 2.83, 104.5) 0.09

Group 3 45.7 15.2 0.146 0.93

Subject 37 3874 104.7

Residual 148 1864 12.6

Cocaine Intake (mg/kg/day)

| Treatment Group | Day 1 | Day 2 | Day 3 | Day 4 | Day 5 | Avg. Days 1-5 |
| --- | --- | --- | --- | --- | --- | --- |
|  |  |  |  |  |  |  |
| NoEXT | 9±1 | 9±1 | 8±1 | 9±1 | 9±1 | 9±1 |
| NoEXT+EE | 11±2 | 10±2 | 9±1 | 10±2 | 10±2 | 10±2 |
| NoEXT+ORG | 8±1 | 9±1 | 8±1 | 8±1 | 8±1 | 8±1 |
| NoEXT+EE+ORG | 9±1 | 8±1 | 9±1 | 8±1 | 9±1 | 9±1 |

**Source of Variation DF SS MS F P**

Group x Session Number 12 15.19 1.27 0.658 0.78

Session Number 4 13.07 3.27 1.697 (df 3.38, 125.1) 0.15

Group 3 91.02 30.34 0.453 0.72

Subject 37 2475.59 66.92

Residual 148 284.80 1.92

**Supplemental Table 2. Self-Administration Behavior During Reacquisition in the Four NoEXT Treatment Groups**

| Treatment Group | Day 1 | Day 2 | Day 3 | Day 4 | Day 5 | Day 6 | Day 7 | Day 8 | Day 9 | Day 10 | Day 11 | Day 12 | Day 13 | Day 14 | Day  15 |  | Days 1-15  AVG |
| --- | --- | --- | --- | --- | --- | --- | --- | --- | --- | --- | --- | --- | --- | --- | --- | --- | --- |
|  |  |  |  |  |  |  |  |  |  |  |  |  |  |  |  |  |  |
| NoEXT | 135  ±35 | 135±38 | 100  ±29 | 106  ±27 | 97  ±18 | 157  ±48 | 149  ±49 | 129  ±39 | 164  ±41 | 129  ±25 | 143  ±22 | 133  ±37 | 139  ±46 | 128  ±29 | 120  ±36 |  | 131±30 |
| NoEXT+EE | 105±12 | 89  ±20 | 107±13 | 145±28 | 141±39 | 86  ±8 | 111±26 | 70  ±10 | 91  ±12 | 93  ±19 | 95  ±15 | 111  ±23 | 84  ±16 | 100±17 | 130±17 |  | 104±9 |
| NoEXT+ORG | 86  ±14 | 100  ±13 | 78  ±25 | 94  ±22 | 115  ±28 | 126±31 | 86  ±23 | 89  ±23 | 79  ±18 | 100±35 | 98  ±29 | 79  ±17 | 75  ±17 | 87  ±25 | 86  ±13 |  | 92±19 |
| NoEXT+EE+ORG | 108±16 | 88  ±7 | 124±46 | 134±48 | 114±28 | 135  ±41 | 171±88 | 165±86 | 133±46 | 108±31 | 203±119 | 121±40 | 127±46 | 116±23 | 127±44 |  | 132±45 |

**Active Lever Responses**

**Source of Variation DF SS MS F P**

Groups x Session Number 42 112128 2670 0.945 0.57

Session Number 14 27915 1994 0.713 (df 3.98, 71.7) 0.59

Group 3 95553 31851 0.444 0.72

Subject 18 1290659 71703

Residual 252 711793 2825

**Inactive Lever Responses**

| Treatment Group | Day  1 | Day  2 | Day 3 | Day 4 | Day 5 | Day 6 | Day 7 | Day 8 | Day 9 | Day 10 | Day 11 | Day 12 | Day 13 | Day 14 | Day  15 |  | Days 1-15  AVG |
| --- | --- | --- | --- | --- | --- | --- | --- | --- | --- | --- | --- | --- | --- | --- | --- | --- | --- |
|  |  |  |  |  |  |  |  |  |  |  |  |  |  |  |  |  |  |
| NoEXT | 5  ±2 | 5  ±2 | 5  ±2 | 3  ±1 | 2  ±2 | 2  ±1 | 2  ±1 | 2  ±1 | 1  ±1 | 2  ±1 | 1  ±1 | 2  ±1 | 3  ±1 | 2  ±1 | 3  ±1 |  | 3±1 |
| NoEXT+EE | 7  ±2 | 2  ±1 | 2  ±1 | 6  ±2 | 4  ±2 | 2  ±1 | 2  ±1 | 2  ±1 | 1  ±1 | 1  ±1 | 1  ±1 | 2  ±1 | 1  ±1 | 1  ±1 | 1  ±1 |  | 2±1 |
| NoEXT+ORG | 5  ±2 | 15  ±10 | 7  ±5 | 6  ±5 | 7  ±5 | 5  ±2 | 3  ±2 | 3  ±2 | 4  ±2 | 1  ±1 | 1  ±1 | 1  ±1 | 2  ±1 | 3  ±1 | 3  ±2 |  | 4±2 |
| NoEXT+EE+ORG | 4  ±1 | 6  ±3 | 3  ±1 | 1  ±1 | 5  ±3 | 7  ±5 | 7  ±4 | 3  ±1 | 4  ±2 | 4  ±3 | 6  ±3 | 5  ±3 | 5  ±3 | 2  ±1 | 6  ±5 |  | 4±2 |

**Source of Variation DF SS MS F P**

Groups x Session Number 42 963.6 22.94 1.317 0.10

Session Number 14 557.8 39.84 2.286 (df 2.55, 45.97) 0.10

Group 3 270.9 90.31 0.456 0.72

Subject 18 3562.0 197.90

Residual 252 4391.0 17.42

| Treatment Group | Day  1 | Day 2 | Day 3 | Day 4 | Day 5 | Day 6 | Day 7 | Day 8 | Day 9 | Day 10 | Day 11 | Day 12 | Day 13 | Day 14 | Day  15 |  | Days 1-15  AVG |
| --- | --- | --- | --- | --- | --- | --- | --- | --- | --- | --- | --- | --- | --- | --- | --- | --- | --- |
|  |  |  |  |  |  |  |  |  |  |  |  |  |  |  |  |  |  |
| NoEXT | 7  ±1 | 7  ±1 | 7  ±1 | 7  ±1 | 7  ±1 | 8  ±2 | 8  ±1 | 7  ±1 | 9  ±1 | 8  ±1 | 9  ±1 | 9  ±1 | 7  ±1 | 8  ±1 | 8  ±1 |  | 8±1 |
| NoEXT+EE | 9  ±3 | 10  ±4 | 11  ±4 | 12  ±3 | 11  ±3 | 10  ±4 | 11  ±3 | 10  ±4 | 11  ±3 | 10  ±4 | 10  ±4 | 12  ±4 | 10  ±4 | 11  ±4 | 11  ±4 |  | 11±4 |
| NoEXT+ORG | 7  ±1 | 8  ±1 | 6  ±1 | 7  ±1 | 7  ±1 | 8  ±1 | 6  ±2 | 6  ±1 | 6  ±1 | 6  ±2 | 7  ±2 | 7  ±1 | 6  ±2 | 6  ±2 | 7  ±1 |  | 7±1 |
| NoEXT+EE+ORG | \| 8  ±2 \| 2 \| 3 \| 4 \| 5 \| 6 \| 7 \| 8 \| 9 \| 10 \| 11 \| 12 \| 13 \| 14 \| 15 \| \| --- \| --- \| --- \| --- \| --- \| --- \| --- \| --- \| --- \| --- \| --- \| --- \| --- \| --- \| --- \| | 7  ±2 | 8  ±2 | 8  ±2 | 8  ±1 | 9  ±2 | 9  ±2 | 8  ±2 | 9  ±2 | 9  ±2 | 9  ±2 | 9  ±2 | 9  ±2 | 9  ±2 | 8  ±2 |  | 9±2 |

**Cocaine Intake (mg/kg/day)**

**Source of Variation DF SS MS F P**

Groups x Session Number 42 85.2 2.00 0.643 0.95

Session Number 14 49.9 3.57 1.146 (df 5.54,99.74) 0.32

Group 3 665.7 221.91 0.712 0.56

Subject 18 5607.4 311.52

Residual 252 785.3 3.12

**Baseline Self-Administration and Extinction Behavior in Male vs. Female Rats**

To determine if females responded differently than males to cocaine self-administration at baseline (prior to initiating treatments) and for active lever responding during EXT training (with or without EE+ORG treatment), additional analyses were conducted. At baseline (Supplemental Fig 1), 2-way ANOVAs (group X sex) were conducted and results showed significant main effects only of sex (females > males) for the number of active lever responses (p≤0.02), number of inactive lever responses (p≤0.02), and daily mg/kg cocaine intake (p≤0.02). These results are consistent with a body of literature showing female rats tend to self-administer more cocaine than male rats (Lynch, 2006).

Despite sex differences in baseline cocaine self-administration behavior, male and female rats in this study behaved similarly to treatment during the 6-week extinction training phase. A 3-way ANOVA (group X sex X session number) was conducted for active lever responding (expressed as percent of baseline). Results showed main and interaction effects only for group and session number (Supplemental Figure 2). Post-hoc Tukey tests of the group X session number interaction showed that during week 2 (p≤0.006) and week 3 (p≤0.001) of extinction training, active lever responding was significantly lower in the male and female groups receiving EE+ORG compared to the male and female groups receiving NoEE+VEH. Thus, the EE+ORG treatment enhanced the rate of extinction learning similarly in male and female rats. Without EE+ORG treatment, extinction learning also was similar in male and female rats albeit at a slower rate compared to EE+ORG treatment. These findings are critical for ruling out differential behavioral effects EE+ORG during extinction training in males vs. females as a source of the differential molecular effects of EE+ORG measured following extinction training (present study) and of the differential cocaine relapse prevention effects of EXT+EE+ORG measured following extinction training in males vs. females trained to self-administer a high 1.0 mg/kg dose of cocaine (Kantak et al., 2020).

**Supplemental Figure 1. Baseline Self-Administration Behavior in Male vs. Female Rats**


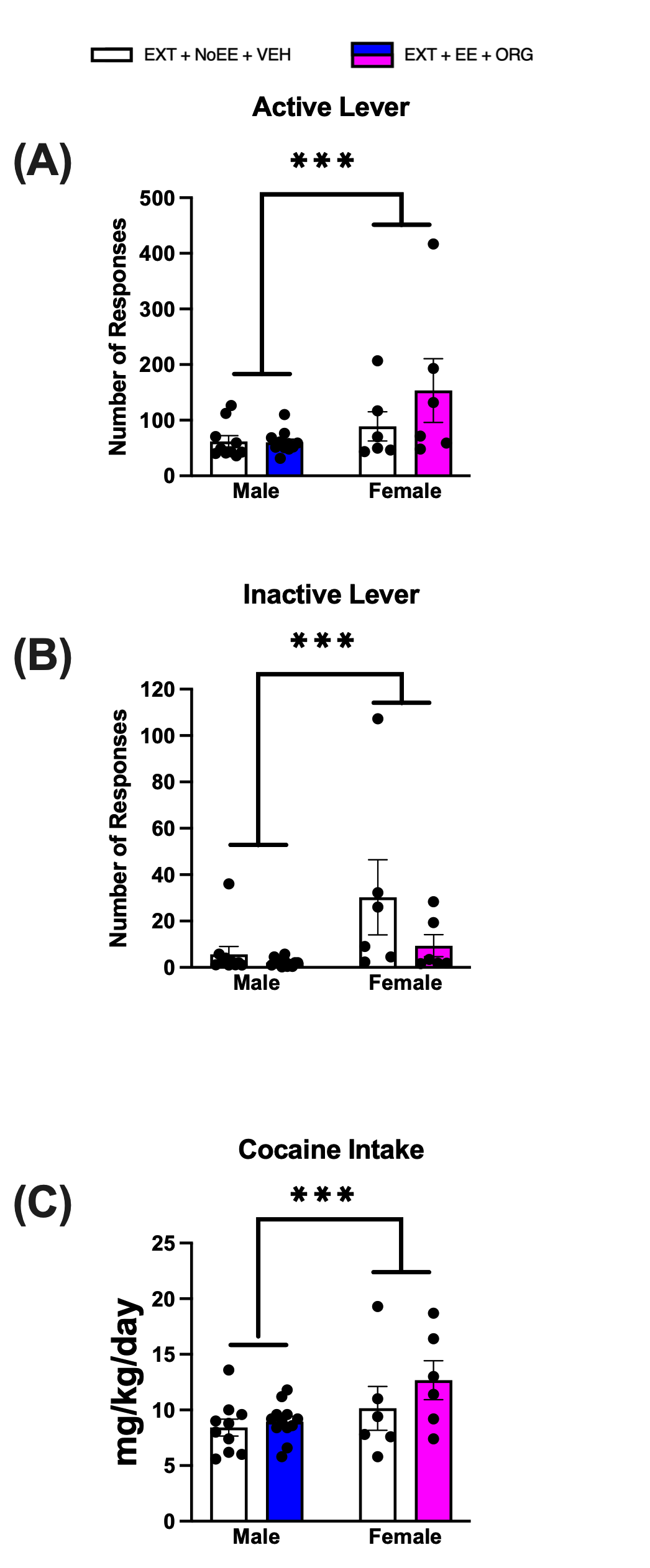


| **Source of Variation**  Group x Sex  Sex  Group  Residual | **DF**  1 | **SS**  8480 | **MS**  8480 | **F**  1.928 | **P**  0.1752 |
| --- | --- | --- | --- | --- | --- |
|  | 1 | 27890 | 27890 | 6.341 | **0.0174** |
|  | 1 | 7611 | 7611 | 1.730 | 0.1983 |
|  | 30 | 131941 | 4398 |  |  |

| **Source of Variation**  Group x Sex  Sex  Group  Residual | **DF**  1 | **SS**  580.6 | **MS**  580.6 | **F**  1.820 | **P**  0.1875 |
| --- | --- | --- | --- | --- | --- |
|  | 1 | 1960 | 1960 | 6.141 | **0.0191** |
|  | 1 | 1154 | 1154 | 3.615 | 0.0669 |
|  | 30 | 9573 | 319.1 |  |  |

| Source of Variation  Group x Sex  Sex  Group  Residual | **DF**  1  1  1  30 | **SS**  7.77  57.77  18.16  289.2 | **MS**  7.77  57.77  18.16  9.642 | **F**  0.8056  5.992  1.884 | **P**  0.3766  **0.0204**  0.1801 |
| --- | --- | --- | --- | --- | --- |

**Supplemental Figure 2. Extinction Behavior in Male vs. Female Rats**

| **Source of Variation**  Session Number | **DF**  5 | **SS**  64860 | **MS**  12972 | **F**  6.129 (df 3.49, 146.6) | **P**  **0.0003** |
| --- | --- | --- | --- | --- | --- |
| Sex | 1 | 36.07 | 36.07 | 0.0034 | 0.9535 |
| Treatment | 1 | 122613 | 122613 | 11.71 | **0.0014** |
| Sex x Session Number | 5 | 5521 | 1104 | 0.5217 | 0.7597 |
| Group x Session Number | 5 | 61147 | 12229 | 5.778 | **0.0001** |
| Group x Sex | 1 | 676.5 | 676.5 | 0.0646 | 0.8006 |
| Group x Sex x Session Number | 5 | 3313 | 662.7 | 0.3131 | 0.9048 |
| Subject | 42 | 439721 | 10470 |  |  |
| Residual | 210 | 444455 | 2116 |  |  |


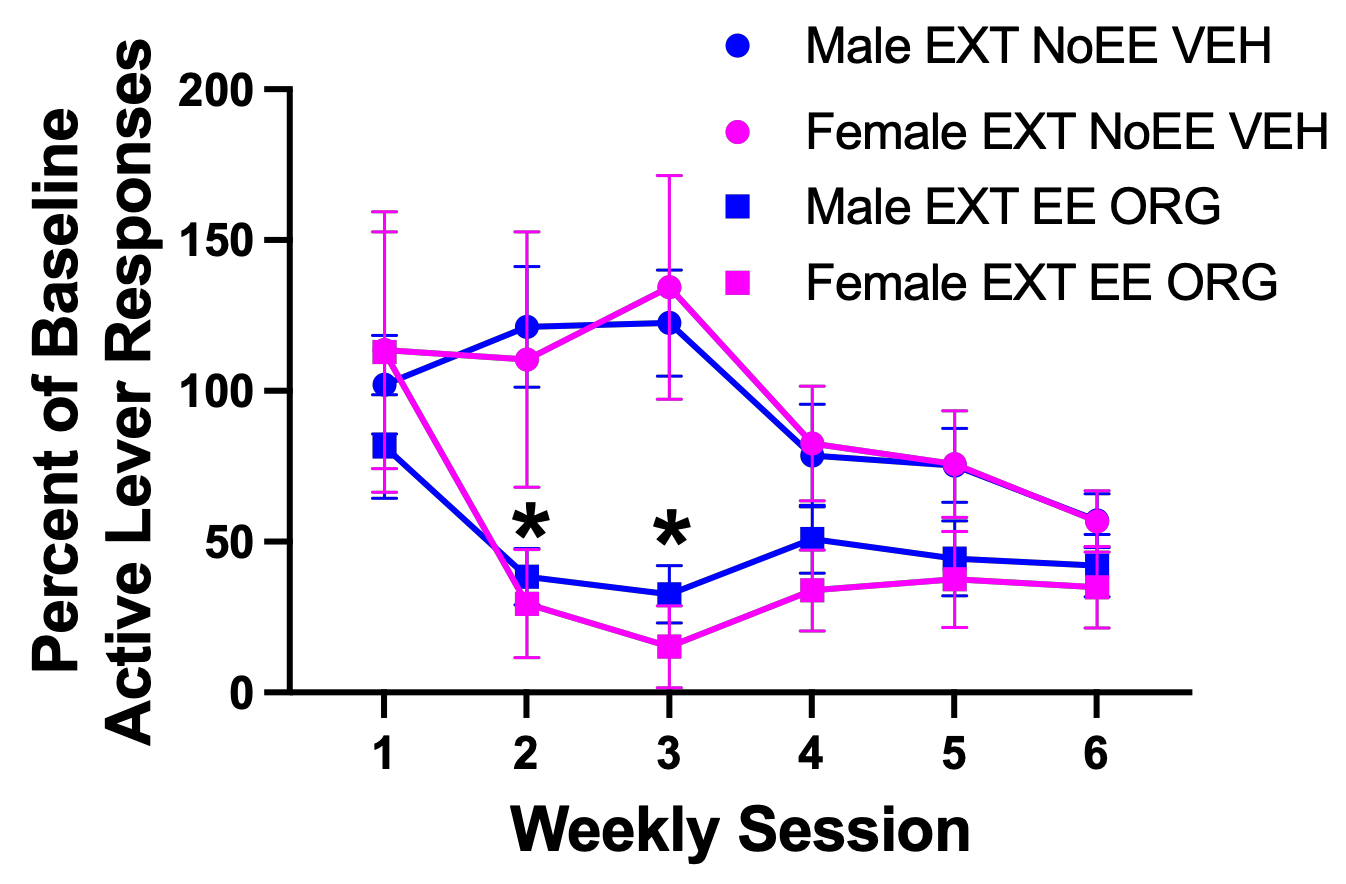


**References**

Kantak, K. M., Gauthier, J. M., Mathieson, E., Knyazhanskaya, E., Rodriguez-Echemendia, P., and Man, H.-Y. (2020). Sex differences in the effects of a combined behavioral and pharmacological treatment strategy for cocaine relapse prevention in an animal model of cue exposure therapy. *Behavioural Brain Research* *395*, 112839. https://doi:10.1016/j.bbr.2020.112839

Lynch, W. J. (2006). Sex differences in vulnerability to drug self-administration. Experimental and Clinical Psychopharmacology, 14(1), 34–41. https://doi.org/10.1037/1064-1297.14.1.34
